# Integration of Single‐Atom Catalyst with *Z*‐Scheme Heterojunction for Cascade Charge Transfer Enabling Highly Efficient Piezo‐Photocatalysis

**DOI:** 10.1002/advs.202303448

**Published:** 2023-08-06

**Authors:** Wenbin Jiang, Hui Zhu, Jing Yang, Beverly Qian Ling Low, Wen‐Ya Wu, Mingxi Chen, Jun Ma, Ran Long, Jingxiang Low, Houjuan Zhu, Jerry Zhi Xiong Heng, Karen Yuanting Tang, Casandra Hui Teng Chai, Ming Lin, Qiang Zhu, Yong‐Wei Zhang, Dongzhi Chi, Zibiao Li, Xian Jun Loh, Yujie Xiong, Enyi Ye

**Affiliations:** ^1^ Institute of Materials Research and Engineering (IMRE) Agency for Science, Technology and Research (A*STAR) 2 Fusionopolis Way, Innovis #08‐03 Singapore 138634 Republic of Singapore; ^2^ Institute of High Performance Computing (IHPC) Agency for Science, Technology and Research (A*STAR) 1 Fusionopolis Way, #16‐16 Connexis Singapore 138632 Republic of Singapore; ^3^ School of Chemistry and Materials Science University of Science and Technology of China Hefei Anhui 230026 P. R. China; ^4^ School of Electrical and Electronic Engineering Nanyang Technological University 50 Nanyang Avenue Singapore 639798 Republic of Singapore; ^5^ Institute of Sustainability for Chemicals, Energy and Environment (ISCE2) Agency for Science, Technology and Research (A*STAR) 1 Pesek Road, Jurong Island Singapore 627833 Republic of Singapore

**Keywords:** cascade charge transfer, molecular oxygen activation, piezo‐assisted photocatalysis, single‐atom catalyst, *Z*‐scheme heterojunction

## Abstract

Piezo‐assisted photocatalysis (namely, piezo‐photocatalysis), which utilizes mechanical energy to modulate spatial and energy distribution of photogenerated charge carriers, presents a promising strategy for molecule activation and reactive oxygen species (ROS) generation toward applications such as environmental remediation. However, similarly to photocatalysis, piezo‐photocatalysis also suffers from inferior charge separation and utilization efficiency. Herein, a *Z*‐scheme heterojunction composed of single Ag atoms‐anchored polymeric carbon nitride (Ag‐PCN) and SnO_2−_ *_x_* is developed for efficient charge carrier transfer/separation both within the catalyst and between the catalyst and surface oxygen molecules (O_2_). As revealed by charge dynamics analysis and theoretical simulations, the synergy between the single Ag atoms and the *Z*‐scheme heterojunction initiates a cascade electron transfer from SnO_2−_ *_x_* to Ag‐PCN and then to O_2_ adsorbed on Ag. With ultrasound irradiation, the polarization field generated within the piezoelectric hybrid further accelerates charge transfer and regulates the O_2_ activation pathway. As a result, the Ag‐PCN/SnO_2−_ *_x_* catalyst efficiently activates O_2_ into ·O_2_^−^, ·OH, and H_2_O_2_ under co‐excitation of visible light and ultrasound, which are consequently utilized to trigger aerobic degradation of refractory antibiotic pollutants. This work provides a promising strategy to maneuver charge transfer dynamics for efficient piezo‐photocatalysis by integrating single‐atom catalysts (SACs) with *Z*‐scheme heterojunction.

## Graphical abstract

Single‐atom catalysts (SACs) are integrated with *Z*‐scheme heterojunction to overcome the fundamental limitation in coupling solar and mechanical energy into oxygen activation (i.e., piezo‐assisted photocatalysis) for environmental remediation. The synergy between single atoms and *Z*‐scheme heterojunction stimulates cascade charge transfer, whereas the material piezoelectric effect regulates the oxygen activation pathway.

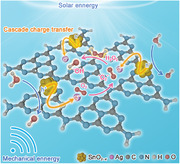

## 1 Introduction

Solar‐driven activation of small molecules (e.g., O_2_ and H_2_O) through photocatalysis presents a sustainable pathway for reactive oxygen species (ROS) generation toward various applications such as environmental remediation.^[^ ^1^ ^]^ More recently, piezo‐photocatalysis that combines the material piezoelectric effect with photocatalysis has emerged as a promising technique for better utilization of solar energy.^[^ ^2^ ^]^ The polarization field generated in the piezoelectric material modulates the spatial and energy distribution of photogenerated charge carriers, facilitating molecule activation and ROS generation.^[^ ^3^ ^]^ Despite the potential, piezo‐photocatalysis has faced a grand challenge similarly to photocatalysis—the sluggish charge transfer dynamics. Before participating in the surface reaction, charge carriers have to be transferred from the bulk to the surface of the catalyst, then to molecules adsorbed on catalyst surface sites. During the processes, most of the charge carriers are recombined, leading to inferior charge utilization efficiency.^[^ ^4^ ^]^ Hence, it is of utmost importance to design catalysts with well‐engineered charge transfer dynamics and surface active sites in order to realize efficient piezo‐photocatalytic molecule activation.

Polymeric carbon nitride (PCN), as a promising organic semiconductor material, has been extensively studied in photocatalysis because of its visible‐light response, reasonable energy band structures, low cost, and high stability.^[^ ^5^ ^]^ In addition, PCN has recently attracted attention as an emerging piezoelectric material due to its superimposed polar tri‐s‐triazine units.^[^ ^6^ ^]^ As such, the rational design of PCN‐based materials (e.g., metal loading, defect engineering, and heterojunction constructing) presents a promising strategy for efficient piezo‐photocatalysis.^[^ ^7^ ^]^ Among the various designs, the construction of the direct *Z*‐scheme heterojunction comprising a reduction photocatalyst (RP, typically PCN) and an oxidation photocatalyst (OP, e.g., inorganic metal oxides and metal sulfides) has drawn wide interest.^[^ ^8^ ^]^ In the direct *Z*‐scheme heterojunction, the photogenerated electrons with strong reduction capability and holes with strong oxidation capability are preserved in the conduction band (CB) of RP and the valence band (VB) of OP, respectively. Electrons in the CB of OP and holes in the VB of RP with weaker redox power recombine through the interfaces of the two photocatalysts.^[^ ^9^ ^]^ Therefore, electrons and holes are efficiently separated within the *Z*‐scheme heterojunction, while retaining their superior redox capabilities for surface catalysis.^[^ ^10^ ^]^

The introduction of surface‐active sites can also be effective in optimizing charge transfer dynamics by strengthening the interaction between catalyst and reactant molecules. In this regard, single‐atom catalysts (SACs) containing atomically dispersed metal sites on catalyst support have been widely investigated.^[^ ^11^ ^]^ Compared to metal nanoparticles, the isolated metal atoms allow substantially enhanced metal‐atom utilization efficiency, while at the same time possessing unique geometric and electronic structures for molecule activation.^[^ ^12^ ^]^ In addition, SACs with clear structures provide an ideal platform for unraveling the relationship between the catalyst structure, charge transfer dynamics, and surface molecule activation mechanism.^[^ ^11c^ ^]^ Inspired by these advantages, integrating SACs with *Z*‐scheme heterojunction is believed to be a feasible pathway to realize subtle control over charge transfer dynamics for efficient piezo‐photocatalysis. The simultaneous in‐plane (single atoms) and out‐of‐plane (heterojunction) modifications can potentially alter both photo‐ and piezoresponses of the material,^[^ ^13^ ^]^ better contributing to the piezo‐assisted photocatalytic process.

Herein, we have developed a *Z*‐scheme heterojunction (Ag‐PCN/SnO_2−_ *_x_*) by integrating single Ag atoms‐anchored PCN (Ag‐PCN) with SnO_2−_ *_x_*, which achieves highly efficient piezo‐photocatalytic O_2_ activation for ROS generation and environmental pollutant degradation. Based on charge dynamics analysis and theoretical simulations, it is revealed that direct contact between Ag‐PCN and SnO_2−_ *_x_* allows a *Z*‐scheme charge transfer under light irradiation which results in electron accumulation in the CB of Ag‐PCN. The accumulated electrons can be transferred to O_2_ through the Ag sites due to the preferential adsorption of O_2_ on Ag, giving birth to a cascade charge transfer. With ultrasound irradiation, the polarization field generated in the piezoelectric hybrid further accelerates the charge transfer and regulates the O_2_ activation pathway. As such, O_2_ molecules are effectively activated into superoxide radical anions (·O_2_^−^), hydroxyl radicals (·OH) and hydrogen peroxide (H_2_O_2_) over Ag‐PCN/SnO_2−_ *_x_* under the co‐irradiation of visible light and ultrasound, successfully triggering the aerobic degradation of refractory antibiotic pollutants. The apparent rate constant of piezo‐photocatalytic degradation reaches 0.0116 min^−1^ for Ag‐PCN/SnO_2−_ *_x_*, 2.4 times that of the PCN/SnO_2−_ *_x_ Z*‐scheme heterojunction. Its performance is further enhanced by 1.5 times to 0.0177 min^−1^ by constructing a piezo‐photocatalytic self‐Fenton system using the in situ produced H_2_O_2_ and externally added trace Fe^3+^.

## 2 Results and Discussion

**Figure 1:**
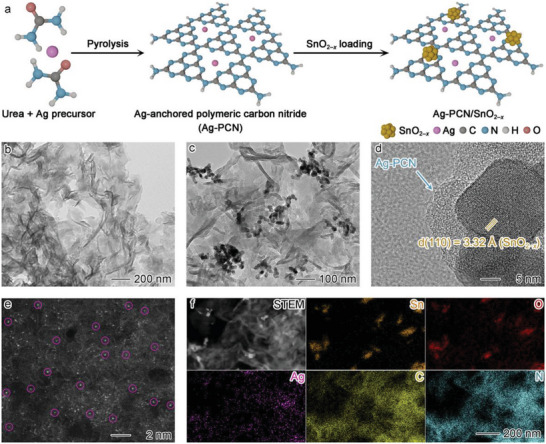
a) Schematic illustration for the synthetic process of Ag atoms‐anchored polymeric carbon nitride (Ag‐PCN)/SnO_2−_ *_x_*. b) Transmission electron microscopy (TEM) image of Ag‐PCN. c) TEM and d) high‐resolution TEM (HRTEM) images of Ag‐PCN/SnO_2−_ *_x_*. e) The spherical aberration‐corrected high‐angle annular dark‐field scanning TEM (HAADF–STEM) image of Ag‐PCN/SnO_2−_ *_x_*. The purple circles highlight some of the single Ag atoms (bright dots). f) STEM image of Ag‐PCN/SnO_2−_ *_x_* and the corresponding energy‐dispersive X‐ray spectroscopy (EDS) elemental mapping profiles showing Sn (orange), O (red), Ag (purple), C (yellow), and N (blue) distributions.

The Ag‐PCN/SnO_2−_ *_x_* catalyst is prepared by assembling presynthesized Ag‐anchored PCN (Ag‐PCN) and SnO_2−_ *_x_* in ethanol (**Figure** **1**a). The Ag‐PCN sample is obtained by one‐pot thermal polymerization of urea and AgNO_3_. In the hybrid catalyst, PCN serves as the antenna to harvest both light and mechanical energy. The suitable energy band alignment between PCN and SnO_2−_ *_x_* is beneficial for *Z*‐scheme charge transfer and visible‐light‐driven catalysis.^[^ ^14^ ^]^ Meanwhile, Ag is selected to modify the PCN surface because of its high conductivity and potential interactions with O_2_.^[^ ^15^ ^]^ The abundant intrinsic nitrogen atoms on PCN enable the atomic dispersion of Ag sites via Ag–N coordination interaction.^[^ ^16^ ^]^

As shown in transmission electron microscopy (TEM) images (Figure 1b and Figure S1, Supporting Information), both PCN and Ag‐PCN samples display thin nanosheet morphology. After SnO_2−_ *_x_* modification (Figure 1c), nanoparticles with an average diameter of ≈14 nm appear on the nanosheet surface (Figure S2, Supporting Information). In the high‐resolution TEM (HRTEM) image (Figure 1d), the lattice fringes with a distance of 3.32 Å can be ascribed to the (110) planes of SnO_2−_ *_x_*. Meanwhile, the graphene‐like substrate is assigned to Ag‐anchored PCN. Since no Ag nanoparticle is observed on the Ag‐PCN surface (Figure S3, Supporting Information), spherical aberration‐corrected high‐angle annular dark‐field scanning TEM (HAADF–STEM) is employed to inspect the status of Ag in the hybrid. As shown in Figure 1e, the bright dots can be identified as single Ag atoms, indicating the atomic dispersion of Ag on the material surface (see also Figure S4, Supporting Information). The N K‐edge electron energy loss spectroscopy (EELS) spectra show that the Ag anchoring is accompanied by an increase in the electron density of N atoms on PCN (Figure S5, Supporting Information), indicating the strong Ag–N interactions between the single Ag atoms and PCN.^[^ ^17^ ^]^ Energy‐dispersive X‐ray spectroscopy (EDS) mapping profiles show that the Ag is homogeneously distributed on the PCN surface, serving as the support for SnO_2−_ *_x_* loading (Figure 1f). The close contact between SnO_2−_ *_x_* and the Ag‐PCN support is beneficial for electronic interactions between the two components. Further characterizations using X‐ray diffraction (XRD), Fourier transform infrared (FTIR) and N_2_ adsorption–desorption isotherms suggest that both Ag and SnO_2−_ *_x_* modifications have no evident influence on the structure of PCN (Figure S6, Supporting Information).

**Figure 2:**
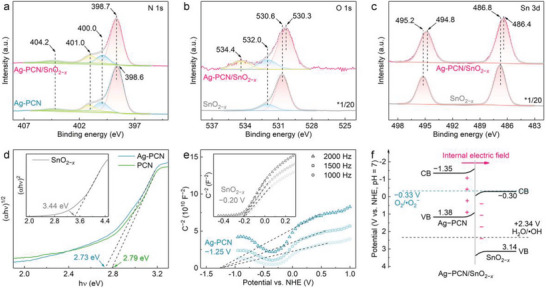
High‐resolution a) N 1s, b) O 1s, and c) Sn 3d X‐ray photoelectron spectroscopy (XPS) spectra for Ag atoms‐anchored polymeric carbon nitride (Ag‐PCN)/SnO_2−_ *_x_*, Ag‐PCN and SnO_2−_ *_x_* samples. d) Tauc plots and e) Mott–Schottky plots for Ag‐PCN and SnO_2−_ *_x_*. f) Schematic illustration for the energy band structure of Ag‐PCN/SnO_2−_ *_x_*, highlighting the formation of an internal electric field.

The electronic interactions between Ag‐PCN and SnO_2−_ *_x_* are studied by X‐ray photoelectron spectroscopy (XPS). In the N 1s spectra (**Figure** **2**a), typical peaks at 400.0, 401.0, and 404.2 eV are ascribed to three‐coordinate N–(C)_3_, –NH_2_ groups and π‐excitation of C–N heterocycles, respectively. Meanwhile, the peaks at 398.6 and 398.7 eV are indexed to two‐coordinate N species (N–C=N).^[^ ^18^ ^]^ The slight positive shift in the N 1s peak after SnO_2−_ *_x_* loading suggests the interfacial charge transfer between Ag‐PCN and SnO_2−_ *_x_*.^[^ ^19^ ^]^ In addition, in the O 1s spectra (Figure 2b), the peaks at 532.0 and 534.4 eV are attributed to chemisorbed oxygen species on SnO_2−_ *_x_* and surface‐adsorbed O_2_ molecules on Ag‐PCN, respectively.^[^ ^20^ ^]^ Notably, after heterojunction formation between SnO_2−_ *_x_* and Ag‐PCN, the binding energy of lattice oxygen in SnO_2−_ *_x_* exhibits a 0.3 eV negative shift from 530.6 to 530.3 eV. A 0.4 eV negative shift is also observed for Sn 3d signals (Figure 2c), verifying the interfacial electron transfer from Ag‐PCN to SnO_2−_ *_x_* (Figure S7, Supporting Information). Beyond that, in the Ag 3d spectra (Figure S8, Supporting Information), the binding energy of Ag 3d_2/5_ at 368.1 eV suggests that Ag is partially positively charged, consistent with the previous report of N‐coordinated single Ag atoms.^[^ ^15b^ ^]^

The electronic band structures of the samples are studied by UV–Vis diffuse reflectance absorption spectroscopy (DRS) and Mott–Schottky measurements. The extended light absorption of SnO_2−_ *_x_* in the visible‐light range corroborates the existence of oxygen vacancies in the material (Figure S9, Supporting Information).^[^ ^20a^ ^]^ Compared to PCN, the Ag‐PCN displays a slight red shift in the absorption edge, indicating a narrowed bandgap (*E*_g_) after Ag anchoring. Based on the Tauc plot (Figure 2d), the *E*_g_ values for PCN, Ag‐PCN and SnO_2−_ *_x_* are determined to be 2.79, 2.73, and 3.44 eV, respectively. In the Mott–Schottky plots (Figure 2e), the positive slopes for PCN, Ag‐PCN and SnO_2−_ *_x_* suggest that all these samples are n‐type semiconductors, with their flat‐band potentials (*E*_fb_) determined to be −1.33, −1.25, and −0.20 V versus NHE (pH = 7), respectively. Given that the CB minimum (*E*_CBM_) is located at about −0.1 V to the *E*_fb_ for n‐type semiconductors,^[^ ^13a^ ^]^ the *E*_CBM_ of PCN, Ag‐PCN and SnO_2−_ *_x_* are estimated to be −1.43, −1.35, and −0.30 eV, respectively. Based on the information gleaned above (*E*_CBM_, *E*_g_, and the interfacial charge transfer), the band structure of the heterojunction can be depicted (Figure 2f, Figure S10, Supporting Information). Specifically, the interfacial electron transfer from Ag‐PCN to SnO_2−_ *_x_* results in the formation of an internal electric field whose direction points from Ag‐PCN to SnO_2−_ *_x_*.^[^ ^9a^ ^]^

**Figure 3:**
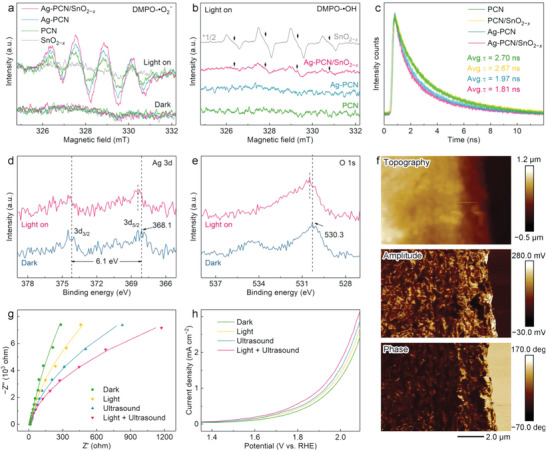
Electron spin resonance (ESR) spectra of a) ·O_2_^−^ and b) ·OH captured using 5,5‐dimethyl‐1‐pyrroline N‐oxide (DMPO) as a spin‐trapping agent. c) Time‐resolved photoluminescence (TRPL) spectra of Ag atoms‐anchored polymeric carbon nitride (Ag‐PCN)/SnO_2−_ *_x_*, Ag‐PCN, PCN/SnO_2−_ *_x_*, and PCN samples. High‐resolution d) Ag 3d and e) O 1s X‐ray photoelectron spectroscopy (XPS) spectra for Ag‐PCN/SnO_2−_ *_x_* in the dark and under light irradiation. f) The piezoresponse force microscopy (PFM) topography of PCN and the corresponding amplitude and phase mapping profiles. g) Electrochemical impedance spectroscopy (EIS) Nyquist plots and the corresponding fitting analysis for Ag‐PCN/SnO_2−_ *_x_* under different conditions. h) Linear sweep voltammetry (LSV) curves for Ag‐PCN/SnO_2−_ *_x_* under different conditions.

The internal electric field, combined with the suitable energy band alignment, is beneficial for the *Z*‐scheme charge transfer under visible‐light irradiation (Figure S11, Supporting Information).^[^ ^21^ ^]^ To verify this argument, electron spin resonance (ESR) tests are carried out using 5,5‐dimethyl‐1‐pyrroline *N*‐oxide (DMPO) as the spin‐trapping agent.^[^ ^22^ ^]^ As shown in **Figure** **3**a, a characteristic 1:1:1:1 quartet signal of DMPO‐trapped ·O_2_^−^ (DMPO‐·O_2_^−^) appears for all the PCN‐based samples. No distinct signal is observed for SnO_2−_ *_x_* due to the weak reduction ability of its photogenerated electrons (−0.30 V versus NHE, pH = 7). On the contrary, the characteristic 1:2:2:1 quartet signal of DMPO‐·OH can only be observed for samples containing SnO_2−_ *_x_* (Figure 3b). These results indicate that photogenerated electrons and holes are accumulated on the CB of PCN and VB of SnO_2−_ *_x_*, respectively, corroborating the *Z*‐scheme charge transfer within the heterojunction catalyst. Moreover, the highest ·O_2_^−^ production on Ag‐PCN/SnO_2−_ *_x_* suggests that single Ag atoms and *Z*‐scheme heterojunction can work collaboratively to promote O_2_ activation (see also Figure S12, Supporting Information).

To understand the synergy between Ag and the *Z*‐scheme heterojunction, the charge transfer dynamics of the samples are studied by steady‐state photoluminescence (PL) and time‐resolved PL (TRPL). The PL results show that the simultaneous modification of Ag and SnO_2−_ *_x_* on PCN results in the most diminished PL intensity (Figure S13a, Supporting Information). This observation is consistent with the highest photocurrent response of the Ag‐PCN/SnO_2−_ *_x_*, indicating the Ag and SnO_2−_ *_x_* co‐promoted charge separation on PCN (Figure S13b, Supporting Information). Moreover, as revealed by TRPL (Figure 3c, Figure S14, Supporting Information), the Ag‐PCN displays a shortened average PL lifetime (*τ*_avg_, 1.97 ns) compared to pure PCN (2.70 ns), while the PCN/SnO_2−_ *_x_* has a similar *τ*_avg_ of 2.67 ns. Interestingly, integrating Ag with the PCN/SnO_2−_ *_x_* leads to the shortest *τ*_avg_ of 1.81 ns. By assuming Ag as the electron acceptor, the charge transfer efficiencies (*η*) from PCN and PCN/SnO_2−_ *_x_* to Ag are determined to be 26.9% and 32.3%, respectively (Table S1, Supporting Information).^[^ ^23^ ^]^ Based on these results, it can be clearly demonstrated that the single Ag atoms are capable of accelerating charge transfer/separation within the PCN/SnO_2−_ *_x_ Z*‐scheme heterojunction.

To look into the Ag‐accelerated charge transfer/separation process, synchronous illumination XPS (SI‐XPS) is carried out to investigate the electron transfer pathway.^[^ ^24^ ^]^ As shown in the Ag 3d spectra of Ag‐PCN/SnO_2−_ *_x_* (Figure 3d), the binding energies of Ag 3d_2/3_ and 3d_5/2_ are positively shifted under light irradiation, indicating an increased electron density on Ag.^[^ ^25^ ^]^ Meanwhile, the peak in the O 1s spectra is positively shifted (Figure 3e), indicating the accumulation of positive charges on SnO_2−_ *_x_* (Figure S15, Supporting Information). In the *Z*‐scheme heterojunction, the electron transfer from SnO_2−_ *_x_* to Ag‐PCN results in photogenerated holes‐accumulated SnO_2−_ *_x_*, which is in agreement with the SI‐XPS observation. Moreover, the increased electron density on Ag suggests that electrons on the Ag‐PCN surface can be accumulated on the Ag sites. As such, a directional electron transfer from SnO_2−_ *_x_* to PCN and subsequently to Ag is established. The single Ag atoms serve as an electron pump to direct electron transfer, leading to accelerated charge transfer/separation within the *Z*‐scheme heterojunction.^[^ ^12a^ ^]^

In principle, PCN as a piezoelectric material will generate an internal polarization field under external stress, which can further regulate the spatial and energy distribution of photogenerated charge carriers.^[^ ^4a^ ^]^ Since the piezoelectric effect of PCN originates from the superimposed polar tri‐s‐triazine units and the noncentrosymmetric triangular nanopores,^[^ ^6^ ^]^ both in‐plane (Ag) and out‐of‐plane (SnO_2−_ *_x_*) modifications have the possibility to modify the local asymmetry and piezoresponse of the material.^[^ ^13^, ^26^ ^]^ Piezoresponse force microscopy (PFM) is employed to examine the piezoelectric effect of PCN and the influence brought by Ag and SnO_2−_ *_x_*. The PFM amplitude–voltage butterfly loops and the ≈180° piezoresponse phase‐reversal hysteresis loops verify the piezoelectric properties of PCN‐based samples (Figure S16, Supporting Information). As shown in Figure 3f, the PFM amplitude and phase mapping profiles match well with the topography of PCN samples, confirming the homogeneous piezoelectric feature within the nanosheets. The strength of the material piezoresponse can be quantitatively compared based on the slope of the PFM amplitude–voltage butterfly loop, where the larger slope represents stronger saturation polarization.^[^ ^13b^ ^]^ The results show that both Ag and SnO_2−_ *_x_* modifications affect the piezoresponse of PCN. Compared with PCN (21.7 mV V^−1^), the Ag‐PCN/SnO_2−_ *_x_* shows a larger piezoelectric slope of 27.6 mV V^−1^, indicating its stronger saturation polarization. Further electrochemical impedance spectroscopy (EIS, Nyquist plots) and linear sweep voltammetry (LSV) analysis indicate that the material possesses the lowest charge transfer resistance (*R*_ct_, 57.2 kΩ cm^2^) and highest current density under co‐excitation of light and ultrasound (Figure 3g,h, Figure S17, Supporting Information). These results demonstrate the effectiveness of simultaneous in‐plane and out‐of‐plane modification in controlling the local asymmetry of PCN for enhanced piezoresponse and accelerated charge transfer/separation.^[^ ^13^, ^26^ ^]^

**Figure 4:**
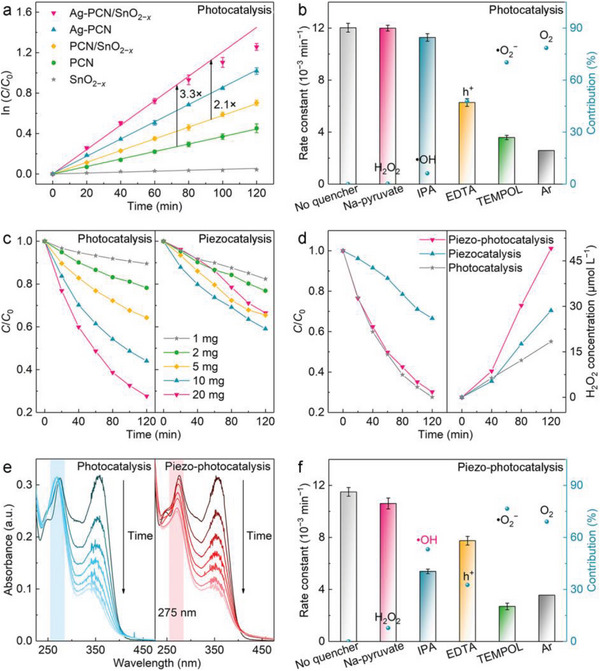
a) Kinetic studies for photocatalytic tetracycline hydrochloride (TCH) degradation over different catalysts. b) Rate constants for photocatalytic TCH degradation over Ag atoms‐anchored polymeric carbon nitride (Ag‐PCN)/SnO_2−_ *_x_* in the presence of different quenchers. c) Photocatalytic and piezocatalytic activities for TCH degradation over Ag‐PCN/SnO_2−_ *_x_* at different catalyst dosages. d) Comparison of photocatalytic, piezocatalytic and piezo‐photocatalytic activities for TCH degradation and H_2_O_2_ production over Ag‐PCN/SnO_2−_ *_x_*. e) Time‐dependent UV–Vis spectra of TCH collected during photocatalytic and piezo‐photocatalytic degradation processes using Ag‐PCN/SnO_2−_ *_x_* as the catalyst, highlighting the different trends for peak evolution at 275 nm. f) Rate constants for piezo‐photocatalytic TCH degradation over Ag‐PCN/SnO_2−_ *_x_* in the presence of different quenchers. The error bars in a), b), and f) represent the standard deviation of three independent experiments.

Upon identifying the charge transfer dynamics within the single Ag atoms‐integrated *Z*‐scheme heterojunction, the performance of the hybrid for molecule activation and ROS generation is evaluated using the degradation of tetracycline hydrochloride (TCH), a typical antibiotic pollutant with biological toxicity,^[^ ^27^ ^]^ as a model reaction. The photocatalytic, piezocatalytic, and piezo‐photocatalytic TCH degradation reactions are carried out under irradiation of light, ultrasound, and the combined light and ultrasound, respectively. The catalytic performance is quantitatively determined based on the characteristic peak of TCH at 357 nm using UV–Vis spectroscopy (Figures S18 and S19, Supporting Information). Under visible light irradiation, the Ag‐PCN/SnO_2−_ *_x_* achieves a degradation rate constant of 0.0121 min^−1^, which is 3.3 and 2.1 times that of PCN and PCN/SnO_2−_ *_x_*, respectively (**Figure** **4**a). This result is in agreement with their capability for visible light driven ·O_2_^−^ production. Moreover, as shown in Figure 4b and Figure S20 (Supporting Information), the catalytic experiments carried out in the presence of different ROS quenchers confirm that ·O_2_^−^ radicals are the main reactive species for photocatalytic TCH degradation.

In piezocatalysis, the performance of Ag‐PCN/SnO_2−_ *_x_* outperforms the other samples, consistent with its strongest piezoresponse (Figure S21, Supporting Information). Noteworthy, the piezocatalytic performance of Ag‐PCN/SnO_2−_ *_x_* decreases at high catalyst dosages (20 mg), in contrast to the phenomenon in photocatalysis (Figure 4c). The reduced piezocatalytic performance at high catalyst dosages can be explained as the increased collision probability between catalysts leads to the quenching effect between positive and negative charges.^[^ ^28^ ^]^ Nonetheless, the piezo‐photocatalytic performance of Ag‐PCN/SnO_2−_ *_x_* increases with increasing catalyst dosages, emphasizing the importance of light energy harvesting in piezo‐photocatalysis (Figures S22 and S23a, Supporting Information). The superior photo‐ and piezoresponses of Ag‐PCN/SnO_2−_ *_x_* bestow it with the highest piezo‐photocatalytic performance (0.0116 min^−1^) among the tested samples, which is 2.4 times that of PCN/SnO_2−_ *_x_* (Figures S23, Supporting Information). The degradation rate constants are similar for photocatalysis and piezo‐photocatalysis using Ag‐PCN/SnO_2−_ *_x_* as the catalyst. However, the H_2_O_2_ production (49.1 µmol L^−1^) during the piezo‐photocatalytic degradation is higher than that of photocatalysis (18.4 µmol L^−1^) and piezocatalysis (28.6 µmol L^−1^) (Figure 4d, Figure S24, Supporting Information). This result indicates that part of charge carriers is consumed for H_2_O_2_ production instead of being engaged in TCH degradation.^[^ ^29^ ^]^

Interestingly, the degradation process monitored via UV–Vis spectra shows a gradually declined peak intensity at 275 nm, indicating the deep removal of TCH during piezo‐photocatalysis (Figure 4e, Figure S25, Supporting Information).^[^ ^30^ ^]^ This phenomenon is hardly observed in the photocatalytic process, suggesting the existence of piezo‐assisted pathways for ROS production and TCH degradation. Compared with photocatalysis, the piezo‐photocatalysis shows enhanced CO production in the gas phase (Figure S26, Supporting Information), further corroborating the piezo‐assisted removal of refractory pollutants. As shown in Figure 4f and Figure S27 (Supporting Information), ·OH radicals, which show negligible contribution (6.2%) in photocatalytic TCH degradation, play an important role (53.1%) in piezo‐photocatalysis. The promoted ·OH production in the piezo‐photocatalytic process is directly evidenced by ESR measurements using DMPO as a spin‐trapping agent (Figure S28, Supporting Information). ·OH radicals possess stronger oxidation capability than ·O_2_^−^ radicals, which explains the success of piezo‐assisted deep removal of TCH. In addition, the contribution of ·O_2_^−^ radicals (76.6%) and h^+^ (32.6%) can be inferred that the majority of ·OH radicals are derived from the reduction of O_2_ (i.e., two‐step O_2_ reduction by electrons),^[^ ^31^ ^]^ rather than the h^+^‐mediated H_2_O oxidation.^[^ ^26a^ ^]^ These results demonstrate the importance of the material piezoelectric effect in regulating the photocatalytic O_2_ activation pathway for promoted ·OH production and refractory pollutant degradation. In addition, cyclic tests show that the performance of Ag‐PCN/SnO_2−_ *_x_* has no significant change during five consecutive tests (Figures S29, Supporting Information). The morphology and structure of Ag‐PCN/SnO_2−_ *_x_* are well maintained after cyclic tests, indicating the excellent stability and reusability of the catalyst (Figure S30, Supporting Information).

It is worth mentioning that the H_2_O_2_ produced in situ is not adequately utilized for pollutant degradation as evidenced by its gradual accumulation in the reaction mixture (Figure 4d). Nonetheless, by introducing a trace amount of Fe^3+^ (10 µmol L^−1^) into the mixture to construct a piezo‐photocatalytic self‐Fenton system,^[^ ^32^ ^]^ the TCH degradation rate is substantially enhanced by 1.5 times from 0.0116 to 0.0172 min^−1^ using Ag‐PCN/SnO_2−_ *_x_* as the catalyst (Figure S31a,b, Supporting Information). In contrast, the photocatalytic performance shows only a slight increase to 0.0123 min^−1^ in the presence of Fe^3+^, demonstrating the superiority of the piezo‐assisted photocatalytic self‐Fenton system for ROS production and pollutant degradation. In addition, a remarkable piezo‐photocatalytic performance (0.0292 min^−1^) is observed over Ag‐PCN/SnO_2−_ *_x_* when simulated sunlight (AM1.5, 15.3 mW cm^−2^) is used as the light source (Figure S31c,d, Supporting Information). The performance of the catalyst in real water systems (natural seawater collected from the east coast, Singapore) is comparable with that in deionized water (Figure S31e,f, Supporting Information). Moreover, other organic pollutants, including ciprofloxacin (CIP), rhodamine B (RhB) and methylene blue (MB) can also be efficiently degraded through piezo‐photocatalysis using Ag‐PCN/SnO_2−_ *_x_* as the catalyst (Figure S32, Supporting Information). The Ag‐PCN/SnO_2−_ *_x_* exhibits a competitive performance with other recently reported piezocatalysts/piezo‐photocatalysts (Table S2, Supporting Information). These results indicate that the Ag‐PCN/SnO_2−_ *_x_* is a promising candidate for practical application.

**Figure 5:**
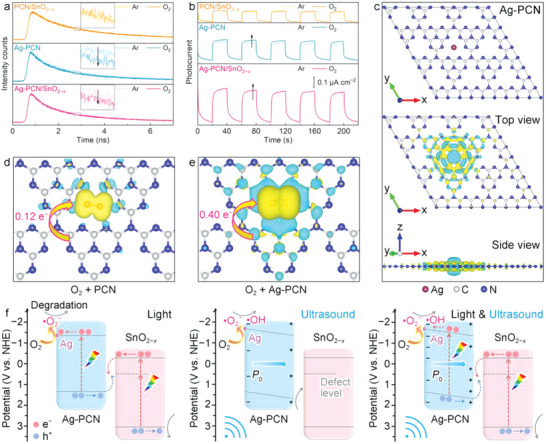
a) Time‐resolved photoluminescence (TRPL) spectra collected in Ar and O_2_ at room temperature for Ag atoms‐anchored polymeric carbon nitride (Ag‐PCN)/SnO_2−_ *_x_*, Ag‐PCN, and PCN/SnO_2−_ *_x_*. The insets show the magnified images of the selected areas in the original curves. b) Transient photocurrent responses for Ag‐PCN/SnO_2−_ *_x_*, Ag‐PCN, and PCN/SnO_2−_ *_x_*. c) Simulated structure model and the corresponding charge density difference for Ag‐PCN. Structure models for O_2_ adsorption on d) PCN and e) Ag‐PCN and the corresponding charge density differences. In c), d), and e), the gray, blue, purple, and red balls represent C, N, Ag, and O atoms, respectively. The yellow and cyan regions represent electron accumulation and electron depletion, respectively. f) Schematic illustration for the charge transfer process and O_2_ activation pathway over Ag‐PCN/SnO_2−_ *_x_* under the excitation of visible light and ultrasound.

To further understand the O_2_ activation process for ROS generation, TRPL measurements are carried out under different atmospheres (Ar and O_2_) to investigate the interaction between O_2_ and the catalyst surface (Figure S33 and Table S3, Supporting Information). Interestingly, the obtained PL decay profiles show that all the Ag‐containing samples (Ag‐PCN and Ag‐PCN/SnO_2−_ *_x_*) display responses to the O_2_ atmosphere (**Figure** **5**a). The *τ*_avg_ of Ag‐PCN/SnO_2−_ *_x_* is shortened from 1.81 to 1.69 ns after switching the atmosphere from Ar to O_2_, while no evident change is observed for PCN/SnO_2−_ *_x_*. Under the O_2_ atmosphere, the Ag‐PCN/SnO_2−_ *_x_* catalyst manifests the shortest *τ*_avg_ of 1.69 ns among all the tested samples (Table S3, Supporting Information). These results strongly suggest that single Ag atoms facilitate charge separation under the O_2_ atmosphere. To validate this feature, transient photocurrent responses are measured in Ar and O_2_.^[^ ^33^ ^]^ As shown in Figure 5b, no distinct difference in the photocurrent density is observed for PCN/SnO_2−_ *_x_*. The Ag‐containing samples display enhanced photocurrent responses when the atmosphere is changed from Ar to O_2_, which corroborates with the Ag‐promoted charge transfer between the catalyst surface and O_2_ molecules.

To gain a deeper understanding of the single Ag atoms‐promoted electron transfer and O_2_ activation process, density functional theory (DFT) calculations are performed for PCN and Ag‐PCN models to explore the potential Ag–O_2_ interactions.^[^ ^15b^ ^]^ The structural model of Ag‐PCN is determined by both experimental and computational observations (Figure 5c). The single Ag atom anchored in the cavity of PCN shows a similar distance of 2.40 Å to the six neighboring N atoms, consistent with the previously reported value.^[^ ^17b^ ^]^ The differential charge density analysis reveals the electron transfer from Ag to the N atoms, accompanied by the formation of a positively charged Ag atom. The net charge of the Ag atom is + 0.60 |e|, which is in agreement with the Ag 3d XPS observation. Furthermore, the O_2_ adsorption on the model surface of PCN and Ag‐PCN is investigated (Figures S34 and S35, Supporting Information). For both model surfaces, the strongest adsorption is that of O_2_ adsorption with a side‐on configuration, and this configuration is henceforth applied for subsequent analysis (Figure 5d,e). The adsorption energies for O_2_ on PCN and Ag‐PCN are calculated to be −0.22 and −0.67 eV, respectively, indicating the Ag‐promoted O_2_ adsorption. In addition, charge density differences suggest that the adsorbed O_2_ captures −0.12 and −0.40 |e| from PCN and Ag‐PCN surfaces, respectively. Compared to free O_2_ molecules (1.21 Å), the O–O bond length is elongated to 1.28 Å for adsorbed O_2_ on the Ag‐PCN surface (Table S4, Supporting Information), close to the reported value of 1.32 Å for ·O_2_^−^ radicals.^[^ ^15b^ ^]^ Compared with PCN, the Ag‐PCN shows enhanced orbital overlapping with the O near the Fermi level, further corroborating the stronger electronic interaction between O_2_ and the Ag‐containing substrate (Figure S36, Supporting Information). These results clearly demonstrate that the single Ag atoms can serve as the active sites for O_2_ adsorption, bridging the electron transfer between the catalyst surface and O_2_ molecules.

Based on the above results and discussion, the synergy between single Ag atoms and the *Z*‐scheme heterojunction in accelerating charge transfer for promoted O_2_ activation and ROS generation can be depicted. As shown in Figure 5f, under visible light irradiation, the *Z*‐scheme charge transfer between Ag‐PCN and SnO_2−_ *_x_* allows energetic electrons and holes to retain in the CB of Ag‐PCN and VB of SnO_2−_ *_x_*, respectively. In the meantime, the preferential adsorption of O_2_ on Ag builds a channel for electron transfer from the Ag‐PCN surface to O_2_ molecules. Consequently, energetic electrons can be utilized to drive the O_2_ activation for ·O_2_^−^ production and the subsequent antibiotic pollutant degradation. In the piezocatalytic process, an internal polarization field (*P*_0_) is generated within the Ag‐PCN/SnO_2−_ *_x_* under ultrasound irradiation. Free electrons and holes are transferred in the opposite direction to participate in reduction and oxidation reactions, respectively, leading to the production of ·O_2_^−^ and ·OH radicals for pollutant degradation. Despite the strong oxidation capability of ·OH, the efficiency of piezocatalysis is limited by the number of effective charge carriers. When the system is co‐irradiated by light and ultrasound, the piezoinduced polarization field within the catalyst assists in the transfer and separation of photogenerated charge carriers. More charge carriers participate in the activation of O_2_ to produce ·O_2_^−^ radicals, H_2_O_2_ and the more oxidative ·OH radicals, contributing to the efficient removal of refractory pollutants. This mechanism elaborates the effectiveness of simultaneous in‐plane (Ag) and out‐of‐plane (SnO_2−_ *_x_*) modification on PCN for efficient piezo‐photocatalysis.

## 3 Conclusion

In summary, we present a single Ag atoms‐integrated PCN/SnO_2−_ *_x_ Z*‐scheme heterojunction for efficient O_2_ activation and ROS generation through piezo‐photocatalysis. The single Ag atoms are stabilized on the PCN surface through Ag–N interactions. Suitable energy band alignment, together with the internal electric field between Ag‐PCN and SnO_2−_ *_x_*, allows the *Z*‐scheme charge transfer under visible light irradiation, leading to electron accumulation on Ag‐PCN. The accumulated electrons can be efficiently transferred to O_2_ molecules due to the preferential adsorption of O_2_ on Ag. Moreover, the piezoresponse of the hybrid allows the formation of an internal polarization field under ultrasound irradiation, further accelerating charge transfer and regulating the O_2_ activation pathway. As a result, O_2_ is efficiently activated into ·O_2_^−^, ·OH, and H_2_O_2_ over Ag‐PCN/SnO_2−_ *_x_* through piezo‐photocatalysis, which successfully triggers aerobic degradation of antibiotic pollutants. The apparent rate constant for degradation reaches 0.0116 min^−1^, 2.4 times that of the Ag‐free heterojunction (PCN/SnO_2−_ *_x_*). The contribution of ·OH radicals is dramatically enhanced from 6.2% in photocatalysis to 53.1% in piezo‐photocatalysis, facilitating the removal of refractory pollutants. The performance is further enhanced by 1.5 times to 0.0177 min^−1^ by constructing a piezo‐photocatalytic self‐Fenton system using the in situ produced H_2_O_2_ and externally added trace amounts of Fe^3+^. This work highlights the effectiveness of piezo‐photocatalysis in environmental remediation, and offers a promising strategy to maneuver charge transfer dynamics for efficient molecule activation using solar and mechanical energy.

## 4 Experimental Section

### Chemicals and Materials

Urea (CO(NH_2_)_2_, 99.0‐100.5%), Tin(II) Chloride (SnCl_2_·2H_2_O, 98.0%), silver nitrate (AgNO_3_, 99.0%), potassium hydroxide (KOH, >85.0%), ethylenediaminetetraacetic acid disodium salt dihydrate (EDTA, 99.0–101.0%), sodium pyruvate (Na‐pyruvate, 99%), 4‐hydroxy‐2,2,6,6‐tetramethylpiperidin‐1‐oxyl (TEMPOL, 97%), TCH (95.0%), MB (1.5%), CIP (98.0%), nitrotetrazolium blue chloride (NBT, 90.0%), DMPO (for ESR spectroscopy), peroxide assay kit (MAK311‐1KT), and Nafion 117 containing solution (≈5% in a mixture of lower aliphatic alcohols and water) were purchased from Sigma–Aldrich. RhB (95.0%) was purchased from TCI. Ethanol and isopropanol (IPA) were purchased from J. T. Baker. All the reagents were used as accepted without further purification. DI water (18.2 MΩ) was used in all the experiments.

### Synthesis of Ag‐Anchored Polymeric Carbon Nitride (Ag‐PCN)

Ag‐PCN samples were prepared by one‐pot thermal polymerization of urea and AgNO_3_. In a typical procedure, urea (30 g) was dissolved in hot water (9 mL, 90 °C), followed by the addition of aqueous AgNO_3_ solution (1 mL, 0.1 m). After stirring for 60 s, the transparent mixture was immediately cooled down using an ice‐water bath, resulting in the formation of solid crystals. After drying in an oven at 70 °C for 12 h, the solid mixture was mortared into powder, followed by sequentially drying at 100 °C for 2 h and calcination at 550 °C for 4 h (heating rate 5 °C min^−1^) in a muffle furnace. The obtained pale yellow Ag‐PCN powder was then collected for further use. The loading amount of Ag in the Ag‐PCN sample was 0.9 wt%. Pure PCN samples were obtained by direct thermal polymerization of urea at 550 °C for 4 h (heating rate 5 °C min^−1^) in a muffle furnace. The yield for all the thermal polymerization processes was about 4 wt% (weight percentage).

### Synthesis of Tin Dioxide Nanoparticles (SnO_2−x_)

SnO_2−_ *_x_* nanoparticles were prepared by modifying a previously reported method.^[^ ^14a^ ^]^ In a typical procedure, SnCl_2_·2H_2_O (6.768 g) was dissolved in a mixture of H_2_O (50 mL) and ethanol (20 mL). Afterward, aqueous KOH solution (30 mL, 2.4 m) was added dropwise into the above solution to form white precipitates. After continuously stirring for 2 h, the precipitate was separated by centrifugation, washed several times with water and ethanol, and dried in an oven at 60 °C overnight. The obtained yellow powder was annealed in Ar at 400 °C for 2 h, followed by calcination in air at 550 °C for 1 h (heating rate 5 °C min^−1^) to produce yellow‐colored SnO_2−_ *_x_*.

### Synthesis of SnO_2−x_ Modified Ag‐PCN (Ag‐PCN/SnO_2−x_)

The Ag‐PCN/SnO_2−_ *_x_* samples were prepared by assembling presynthesized Ag‐PCN and SnO_2−_ *_x_* in ethanol. In a typical procedure, Ag‐PCN (200 mg) was dispersed in ethanol (20 mL) and sonicated for 60 min. Afterward, SnO_2−_ *_x_* nanoparticles‐dispersed ethanol (12 mg, 2 mL) was added into the above suspension. The mixture was sonicated for another 30 min, followed by stirring at 100 rpm for 2 h. After that, the ethanol solvent was removed by drying the mixture at 70 °C for 5 h. Finally, the collected solid powder was annealed in Ar at 400 °C for 1 h (heating rate 2 °C min^−1^) to enhance the interaction between Ag‐PCN and SnO_2−_ *_x_*.

### Material Characterization

Powder XRD patterns were obtained using a Bruker D8 ADVANCE powder diffractometer with Cu‐Kα radiation (*λ* = 1.5418 Å). Scanning electron microscopy (SEM) images were taken on a JEOL JSM‐IT500HR/LV instrument at an accelerating voltage of 5 kV. TEM images were taken on a JEOL JEM‐2100F or an FEI Titan 80–300S/TEM instrument, both operated at 200 kV. HRTEM, scanning TEM (STEM), EDS mapping, and EELS were obtained using the FEI Titan 80–300S/TEM instrument. The spherical aberration‐corrected HAADF–STEM images were taken on a Thermo Scientific Themis Z instrument. XPS was conducted on an Escalab 250 Xi photoelectron spectrometer. All the binding energies were calibrated according to the adventitious carbon located at 284.8 eV. SI‐XPS was performed using a SPECS NAP‐XPS system equipped with a 300 W Xenon lamp as the illumination source. All the XPS spectra were acquired using monochromatized Al Kα (*hν* = 1486.7 eV) as the excitation source. PFM measurements were carried out using atomic force microscopy (AFM, Bruker Dimension Icon) equipped with a PFM module. UV–Vis spectra were taken on a Jasco V‐730 BIO spectrophotometer at a resolution of 0.5 nm. UV–Vis DRS was measured on a Shimadzu UV‐3600 spectrophotometer equipped with an integrating sphere and using BaSO_4_ as the reference. FTIR spectroscopy was performed on a PerkinElmer System 2000 spectrometer in the range of 4000–400 cm^−1^ at a resolution of 6 cm^−1^. N_2_ adsorption–desorption isotherms were measured at 77 K on a Micromeritics ASAP 2020 surface area and porosity analyzer after the samples were degassed at 120 °C for 10 h. The surface area and pore size distribution were calculated by Brunauer–Emmett–Teller (BET) and Barrett–Joyner–Halenda (BJH) methods, respectively. The steady‐state PL spectra were recorded by a Fluoromax‐4 spectrometer (Horiba Scientific) using a nano‐LED (*λ* = 374 nm) as the excitation source. TRPL spectra with a time resolution of 4 ps were collected using a photon‐counting photomultiplier (PMT, PMA‐182, PicoQuant) and processed by a PicoHarp 300 software. A pulse (≈140 fs) femtosecond mode‐locked Ti:sapphire laser (Chameleon Ultra II, Coherent) was used as the excitation source (*λ* = 374 nm). The ESR measurement was performed on a JEOL FA200 ESR spectrometer operated at the X‐band frequency.

### Photoelectrochemical Measurement

Photoelectrochemical (PEC) measurements were performed on an Autolab 302N electrochemical workstation with a standard three‐electrode configuration. A total of 0.2 m Na_2_SO_4_ aqueous solution (pH = 7) was used as the electrolyte and was prepurged with Ar for 15 min to ensure the repeatability of the measurements. A catalyst electrode based on fluorinated tin oxide (FTO) conductive glass, a platinum nanoplate (1 × 1 cm), and a saturated Ag/AgCl electrode (0.1981 V versus NHE) were used as the working electrode, counter electrode and reference electrode, respectively. To prepare the catalyst electrode, as‐prepared catalysts (8.0 mg) were dispersed in IPA (0.5 mL), followed by the addition of 5 µL of 5% Nafion 117. Then, 50 µL of the mixture was withdrawn and drip‐coated onto an FTO glass with an exposed surface area of 1.2 cm^2^. The catalyst electrode was obtained after drying at 40 °C overnight and the catalyst loading is ≈0.6 mg cm^−2^. Transient photocurrent measurements were conducted at an applied potential of 0.5 V (versus Ag/AgCl) with a light on/off interval of 20 s. A 300 W xenon lamp (Perfect Light, PLS‐SXE 300+) was used as the light source for visible light (*λ* > 420 nm) irradiation. Mott–Schottky measurements were carried out at frequencies of 1000, 1500, and 2000 Hz. All the applied potentials were transformed using the equation: *E* (versus NHE) = *E* (versus Ag/AgCl) + 0.1981. For electrochemical measurements carried out under ultrasound irradiation, the catalyst electrodes were prepared by replacing the FTO substrate with carbon cloth. The EIS measurements were conducted in the frequency range from 10^5^ to 0.1 Hz at a sinusoidal ac perturbation of 10 mV. The light and ultrasound were provided by a 300 W xenon lamp (Perfect Light, PLS 300+) and a 280 W ultrasonicator (Elmasonic S 30H), respectively.

### Detection of Hydroxyl Radical (·OH) and Superoxide Radical Anion (·O_2_^−^)

·OH and ·O_2_^−^ radicals were captured using DMPO as a spin‐trapping agent and measured by ESR. In a typical procedure for ·OH detection, samples (5 mg) were dispersed in Ar‐saturated H_2_O (2.5 mL). Then aqueous DMPO solution (30 µL, 2.0 m) was added to the sample suspension, followed by vigorous shaking for 10 s. The mixture was irradiated either by light, ultrasound, or the combined light and ultrasound for 30 s and then analyzed by ESR (JEOL FA200). In a typical procedure for ·O_2_^−^ detection, samples (5 mg) were dispersed in oxygen‐saturated methanol (2.5 mL). Then methanolic DMPO solution (50 µL, 0.2 m) was added to the sample suspension, followed by vigorous shaking for 10 s. The mixture was irradiated either by light, ultrasound, or the combined light and ultrasound for 30 s and then analyzed by ESR (JEOL FA200). The ·O_2_^−^ production was also monitored by NBT degradation. In a typical procedure, samples (2 mg) were dispersed in water (5 mL), followed by the addition of an aqueous NBT solution (125 µL, 1 × 10^−3^ m). The resulting mixture was stirred in the dark for 10 min. Subsequently, a xenon lamp was used as the light source to irradiate the mixture. After a certain time (10 min), 1 mL of the mixture was withdrawn and filtered using a 0.2 µm PTFE filter. The absorption of the filtrate at 259 nm was monitored by a UV–Vis spectrophotometer (Jasco V‐730 BIO).

### Catalytic Experiments for Degradation of Antibiotic

The performance of as‐prepared catalysts for O_2_ activation and ROS generation was evaluated by photocatalytic, piezocatalytic, and piezo‐photocatalytic degradation of TCH. In a typical piezo‐photocatalytic process, catalysts (20 mg) were dispersed in an aqueous solution of TCH (50 mL, 10 mg L^−1^). After stirring in the dark for 30 min to reach adsorption–desorption equilibrium, the suspension was subjected to irradiation of ultrasound (37 kHz) and visible light (*λ* > 420 nm). The ultrasound and visible light were provided by a 280 W ultrasonicator (Elmasonic S 30H) and a 300 W xenon lamp (Perfect Light, PLS 300+), respectively. After every 20 min, 1 mL of the suspension was withdrawn and filtered by a PTFE filter (0.2 µm). The obtained filtrate was analyzed by a UV–Vis spectrophotometer (Jasco V‐730 BIO). The peak intensity at 357 nm was used to calculate the concentration of TCH. In scavenger experiments, 1 × 10^−3^ m of Na‐pyruvate, IPA, TEMPOL, and EDTA was added into the catalytic TCH degradation system, serving as the quencher for H_2_O_2_, ·OH, ·O_2_^−^ and photogenerated holes (h^+^), respectively. A gas chromatograph (GC, 8890, Ar carrier, Agilent) equipped with a flame ionization detector (FID) was used for the determination of CO products in the reaction system. The H_2_O_2_ production in the reaction mixture was monitored using a colorimetric‐based peroxide assay kit (MAK311‐1KT, Sigma–Aldrich). The peak at 585 nm attributed to the Fe^3+^–xylenol orange complex was used to quantify the H_2_O_2_ concentration. The initial concentration of other pollutants (CIP, RhB, and MB) was 10 mg L^−1^. The concentration of these pollutants was monitored using a UV–Vis spectrophotometer. The characteristic peaks for CIP, RhB, and MB are at 272, 553.5, and 664 nm, respectively.

### Theoretical Calculation

The energy and electronic structure calculations were performed with spin‐polarized DFT simulations implemented in the Vienna ab initio simulation package.^[^ ^34^ ^]^ The ionic cores were described using the projected augmented wave (PAW) method. The electronic exchange‐correlation potential was analyzed using the generalized gradient approximation (GGA) of the Perdew–Burke–Ernzerhof (PBE) formulation.^[^ ^35^ ^]^ The energy cutoff of the plane‐wave basis was set to be 500 eV. The force and energy convergence criteria were set to be 0.03 eV Å^−1^ and 10^−5^ eV, respectively. The first Brillouin zone was sampled with a 3 × 3 × 1 k‐point grid using the Monkhorst–Pack scheme. The long‐range van der Waals correction was performed by adopting Grimme's DFT‐D3 method. The adsorption energies (*E*_ads_) of O_2_ were calculated as *E*_ads_ = *E*_total_ − *E*_surf_ − *E*_O2_, where *E*_total_, *E*_surf_, and *E*_O2_ represent the energies of optimized adsorption configurations, empty model surface and gaseous O_2_, respectively.

## Conflict of Interest

The authors declare no conflict of interest.

## Supporting information

Supporting Information

## Data Availability

The data that support the findings of this study are available in the supplementary material of this article.
