# Supplementary material for: Integration of Single‐Atom Catalyst with Z‐Scheme Heterojunction for Cascade Charge Transfer Enabling Highly Efficient Piezo‐Photocatalysis
Source: Adv Sci (Weinh). 2023 Aug 6;10(28):2303448. doi: 10.1002/advs.202303448 (PMC10558689; doi:10.1002/advs.202303448)
Supplement: Supplementary file 1 — Supporting Information [file ADVS-10-2303448-s001.pdf]

ADVANCED SCIENCE
Open Access

Supporting Information

for *Adv. Sci.*, DOI 10.1002/advs.202303448

Integration of Single-Atom Catalyst with *Z*-Scheme Heterojunction for Cascade Charge Transfer Enabling Highly Efficient Piezo-Photocatalysis

Wenbin Jiang, Hui Zhu, Jing Yang, Beverly Qian Ling Low, Wen-Ya Wu, Mingxi Chen, Jun Ma, Ran Long, Jingxiang Low, Houjuan Zhu, Jerry Zhi Xiong Heng, Karen Yuanting Tang, Casandra Hui Teng Chai, Ming Lin, Qiang Zhu, Yong-Wei Zhang, Dongzhi Chi, Zibiao Li, Xian Jun Loh*, Yujie Xiong* and Enyi Ye**

Supporting Information

Integration of Single-Atom Catalyst with Z-Scheme Heterojunction for Cascade Charge Transfer Enabling Highly Efficient Piezo-Photocatalysis

Wenbin Jiang, Hui Zhu, Jing Yang, Beverly Qian Ling Low, Wen-Ya Wu, Mingxi Chen, Jun Ma, Ran Long, Jingxiang Low, Houjuan Zhu, Jerry Zhi Xiong Heng, Karen Yuanting Tang, Casandra Hui Teng Chai, Ming Lin, Qiang Zhu, Yong-Wei Zhang, Dongzhi Chi, Zibiao Li, Xian Jun Loh,* Yujie Xiong,* and Enyi Ye**

Dr. W. Jiang,[+] B. Q. L. Low, Dr. W.-Y. Wu, Dr. M. Chen, Dr. H. Zhu, J. Z. X. Heng, K. Y. Tang, C. H. T. Chai, Dr. M. Lin, Dr. Q. Zhu, Dr. D. Chi, Dr. Z. Li, Dr. X. J. Loh, Dr. E. Ye
Institute of Materials Research and Engineering (IMRE), Agency for Science, Technology and Research (A*STAR), 2 Fusionopolis Way, Innovis #08-03, Singapore 138634, Republic of Singapore
E-mail: lizb@imre.a-star.edu.sg; lohxj@imre.a-star.edu.sg; yeey@imre.a-star.edu.sg

Dr. Z. Li, Dr. X. J. Loh, Dr. E. Ye
Institute of Sustainability for Chemicals, Energy and Environment (ISCE^2), Agency for Science, Technology and Research (A*STAR), 1 Pesek Road, Jurong Island, Singapore 627833, Republic of Singapore

Dr. J. Ma, Prof. R. Long, Dr. J. Low, Prof. Y. Xiong
School of Chemistry and Materials Science, University of Science and Technology of China, Hefei, Anhui, 230026 P. R. China
E-mail: yjxiong@ustc.edu.cn

Dr. J. Yang,[+] Dr. Y.-W. Zhang
Institute of High Performance Computing (IHPC), Agency for Science, Technology and Research (A*STAR), 1 Fusionopolis Way, #16-16 Connexis, Singapore 138632, Republic of Singapore

Dr. H. Zhu[+]
School of Electrical and Electronic Engineering, Nanyang Technological University, 50 Nanyang Avenue, Singapore 639798, Republic of Singapore

[+] These authors contributed equally.

Keywords: piezo-assisted photocatalysis, single-atom catalyst, Z-scheme heterojunction, cascade charge transfer, molecular oxygen activation

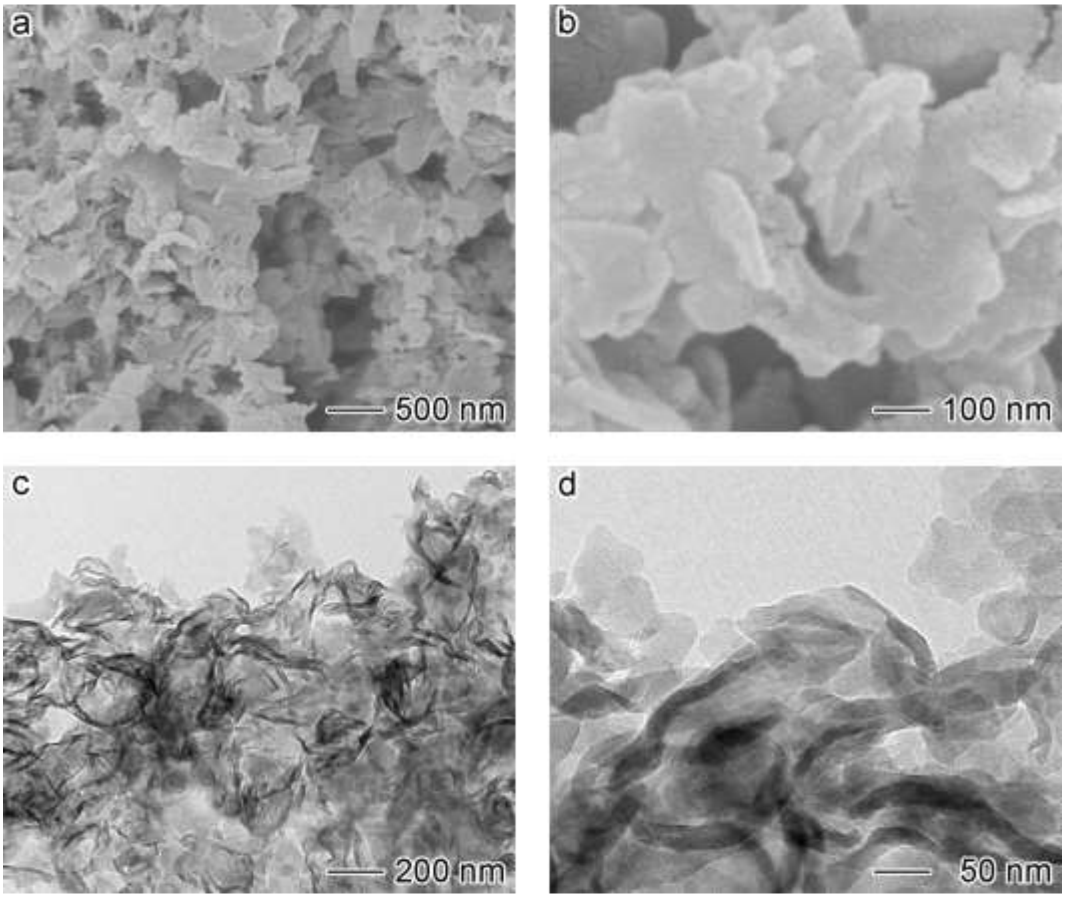

Figure S1. (a,b) SEM and (c,d) TEM images of PCN taken at different magnifications.

As shown in SEM and TEM images (Figure S1), the PCN displays a morphology of two-dimensional (2D) thin nanosheets.

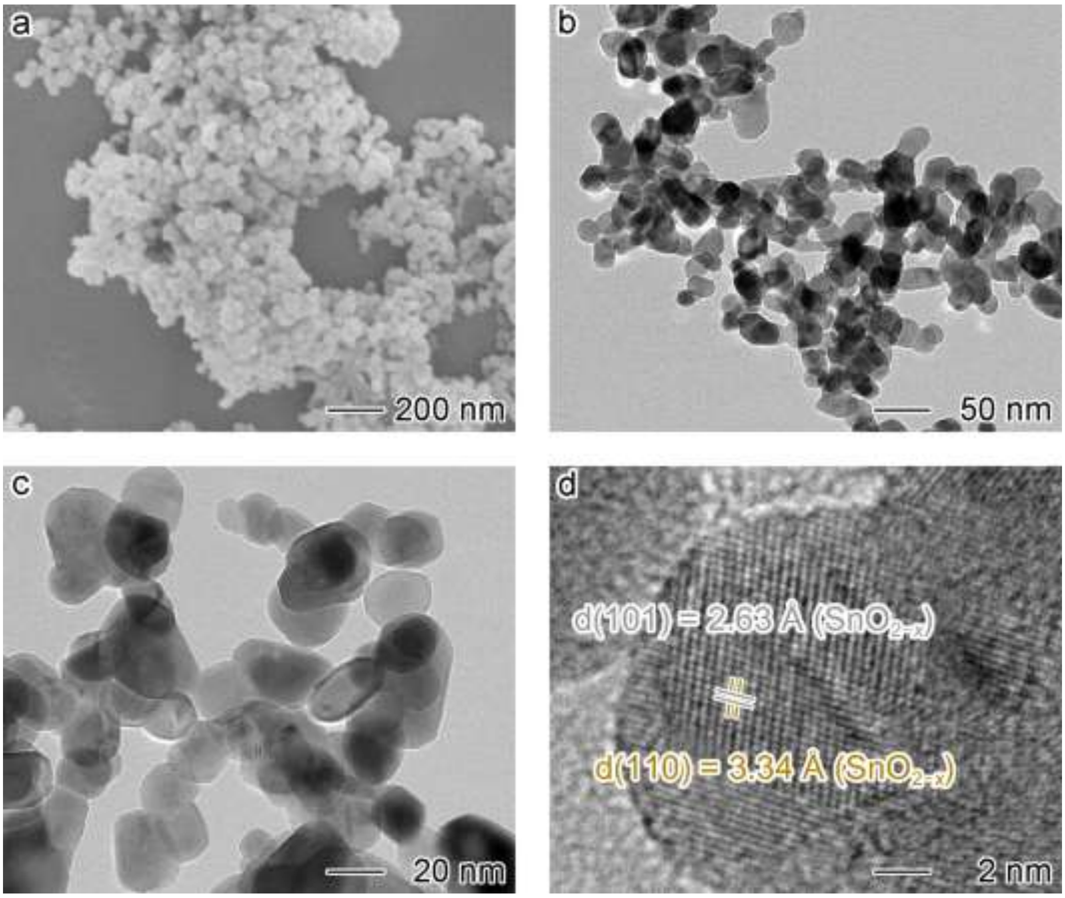

Figure S2. (a) SEM, (b,c) TEM and (d) HRTEM images of SnO_{2-x}.

As shown in SEM and TEM images (Figure S2), the SnO_{2-x} sample displays a nanoparticle morphology with a diameter of about 14 nm. In the HRTEM image (Figure S2d), the ordered lattice fringes with distances of 2.63 and 3.34 Å can be assigned to the (101) and (110) planes of tetragonal SnO_{2-x}, respectively.

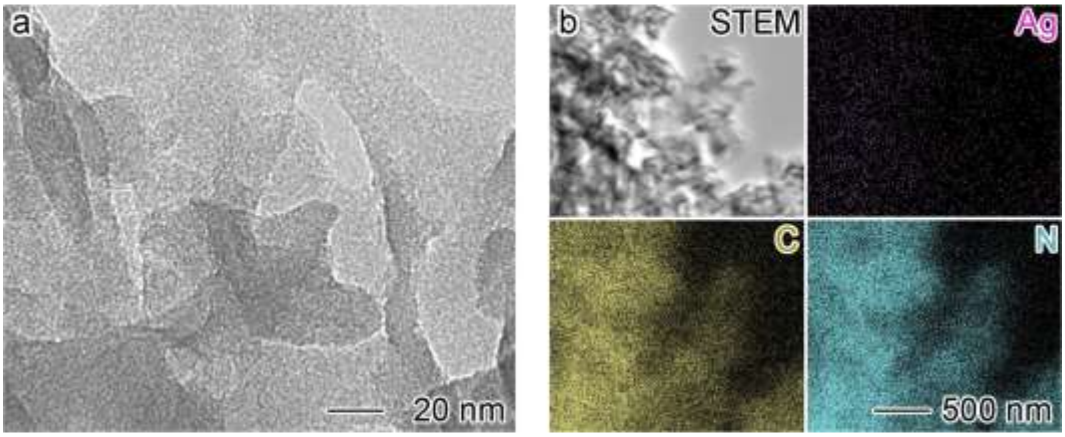

Figure S3. (a) HRTEM image of Ag-PCN. (b) STEM image of Ag-PCN and the corresponding EDS elemental mapping profiles showing Ag (purple), C (yellow) and N (blue) distributions.

As shown in the HRTEM image (Figure S3), no Ag nanoparticles can be observed on the surface of Ag-PCN. EDS mapping profiles and the corresponding STEM image shows the existence of Ag, C and N elements, where Ag is homogeneously dispersed on the sample surface.

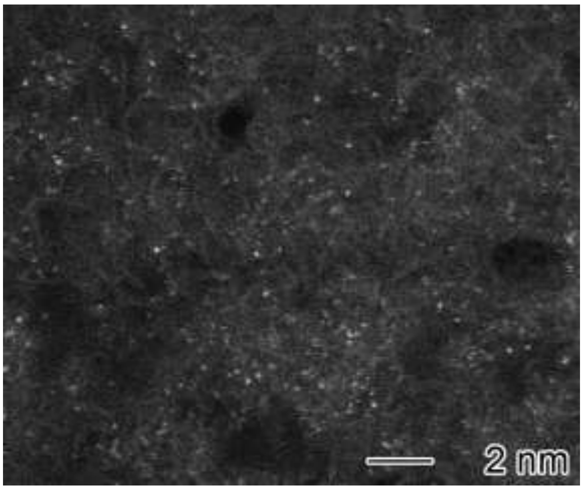

Figure S4. The spherical aberration-corrected HAADF-STEM image of Ag-PCN/SnO_{2-x}.

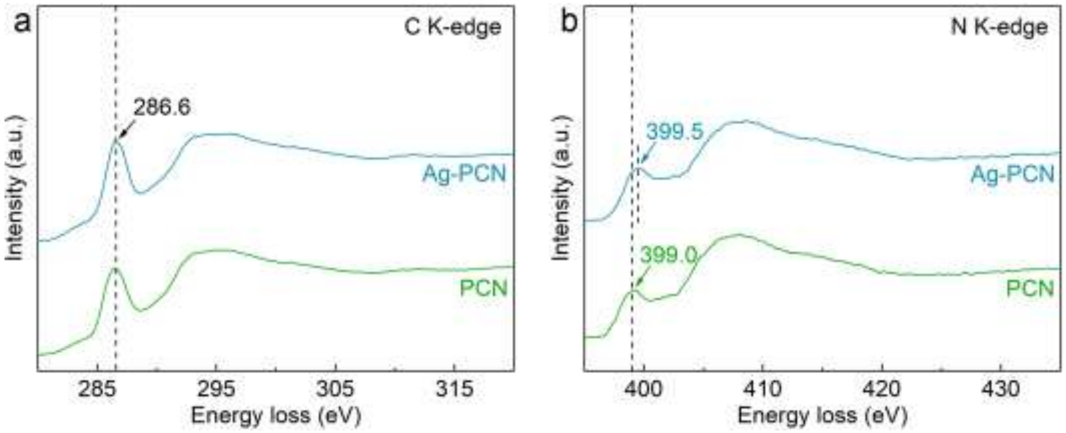

Figure S5. (a) C K-edge and (b) N K-edge EELS spectra of PCN and Ag-PCN.

Electron energy loss spectroscopy (EELS) measurements are carried out to investigate the interaction between Ag and PCN. As shown in the N K-edge spectra (Figure S5), the π* peak at 399.0 eV exhibits a 0.5 eV blue shift after the Ag anchoring, indicating the increased electron density on N atoms.[1] In addition, no obvious change is observed in the C K-edge spectrum. These results suggest that single Ag atoms are stabilized on the PCN substrate through the Ag–N coordination interaction.[2]

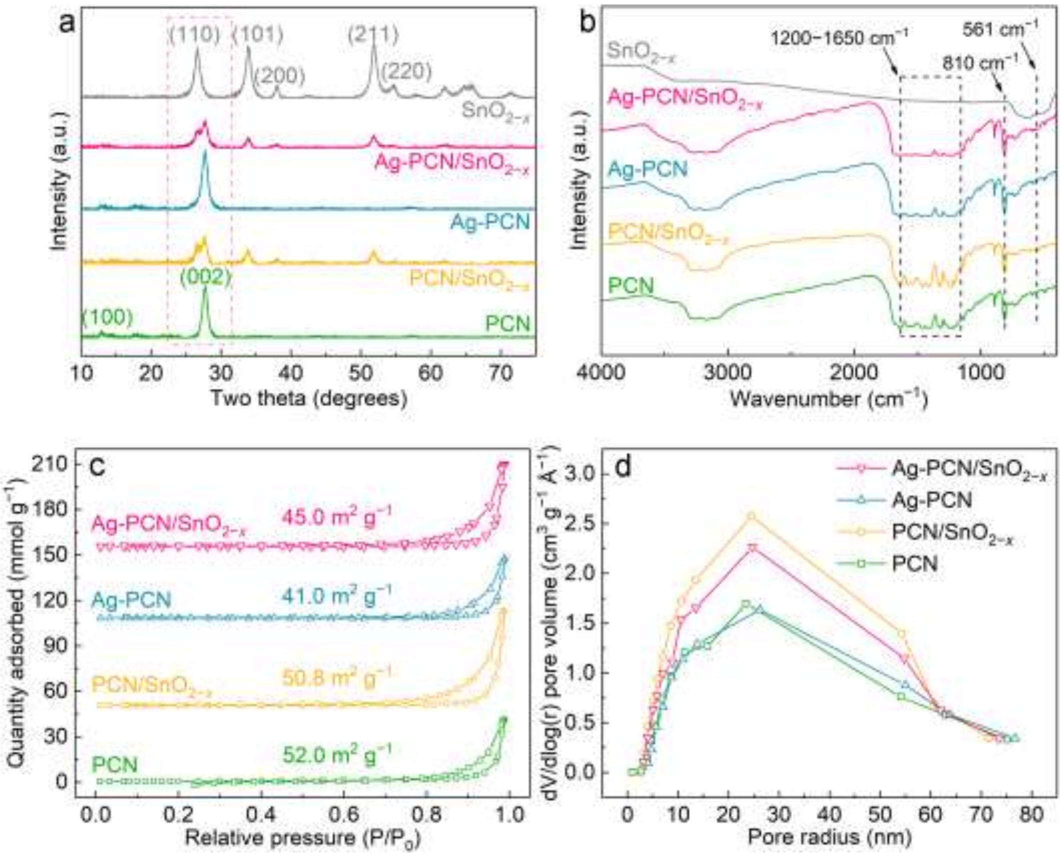

Figure S6. (a) XRD patterns and (b) FTIR spectra of PCN, PCN/SnO_{2-x}, Ag-PCN, Ag-PCN/SnO_{2-x} and SnO_{2-x} samples. (c) N_2 adsorption-desorption isotherms and (d) the calculated pore size distributions for PCN, PCN/SnO_{2-x}, Ag-PCN and Ag-PCN/SnO_{2-x} samples.

Figure S6a shows the XRD patterns of as-prepared samples. For PCN and Ag-PCN, two characteristic diffraction peaks of polymeric carbon nitride appeared at 2θ = 12.9° (100) and 27.7° (002), which can be assigned to the in-plane structural packing of tri-s-triazine units and interlayer stacking of conjugated aromatic frameworks, respectively.[3] The Ag anchoring has no obvious influence on the packing/stacking structure of PCN. After SnO_{2-x} loading, all the XRD peaks of Ag-PCN/SnO_{2-x} can be well indexed to polymeric carbon nitride and the tetragonal phase of Tin dioxide (PDF 41-1445). Figure S6b shows the FTIR of as-prepared samples. For all the PCN-containing samples, the peaks at 1650–1200 and 810 cm^{-1} can be ascribed to the vibrational modes of aromatic C–N heterocycles and tri-s-triazine rings, respectively.[4] The Ag anchoring and SnO_{2-x} loading have no significant influence on the molecular structure of PCN. In the FTIR spectra of Ag-PCN/SnO_{2-x}, the peak at 561 cm^{-1} can be assigned to the characteristic peak of SnO_{2-x}.[5] Figure S6c shows the N_2 adsorption–desorption isotherms for PCN, PCN/SnO_{2-x}, Ag-PCN and Ag-PCN/SnO_{2-x} samples. The isotherms for all the samples display the typical type IV isotherm curve with an H3 hysteresis

loop, indicating the slit-shaped mesoporous characteristics. The surface areas and pore size distributions of the samples are calculated according to Brunauer–Emmett–Teller (BET) and Barrett–Joyner–Halenda (BJH) methods, respectively. The BET surface areas for PCN, PCN/SnO_{2-x}, Ag-PCN and Ag-PCN/SnO_{2-x} samples are determined to be 52.0, 50.8, 41.0 and 45.0 m^2 g^{-1}, respectively. All the samples display similar pore size distributions with a maximum of ~25 nm (Figure S6d).

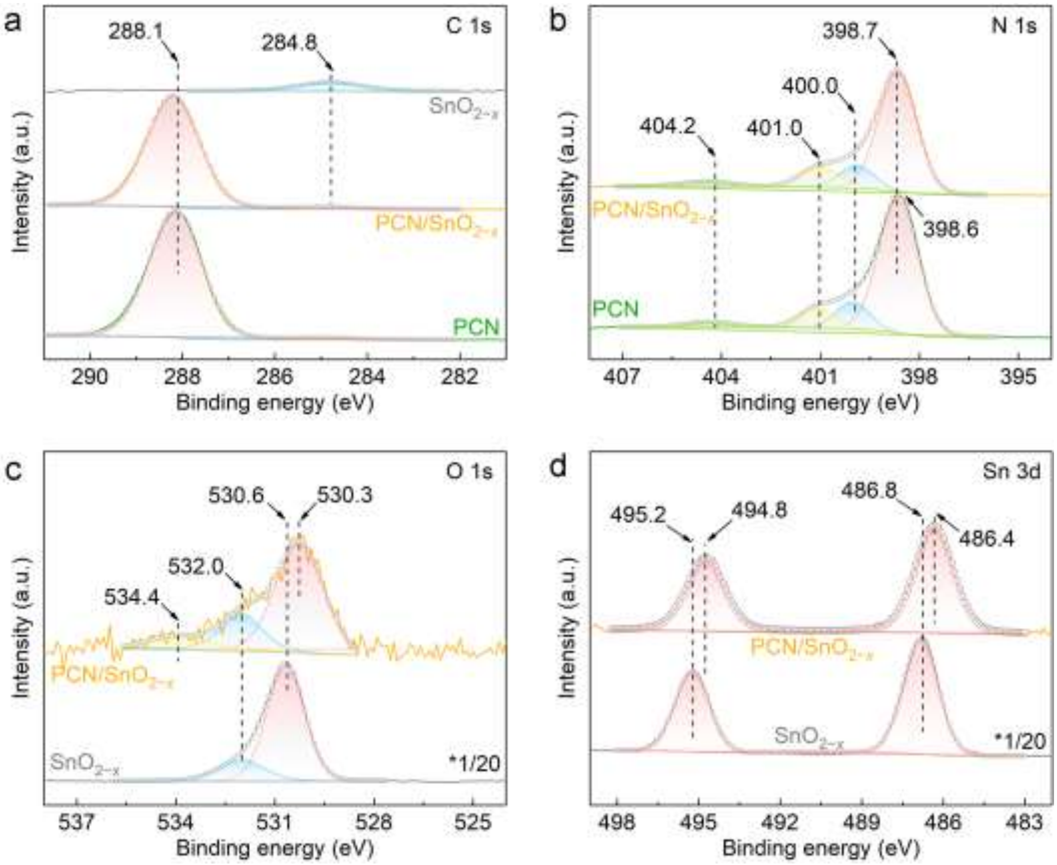

Figure S7. High-resolution (a) C 1s, (b) N 1s, (c) O 1s and (d) Sn 3d XPS spectra for PCN/SnO_{2-x}, PCN and SnO_{2-x} samples.

According to the XPS results (Figure S7), the charge transfer behaviors within the PCN/SnO_{2-x} heterojunction are similar to that of Ag-PCN/SnO_{2-x} (Figure 2a–c). This result suggests that the heterojunction formation is independent of the Ag modification.

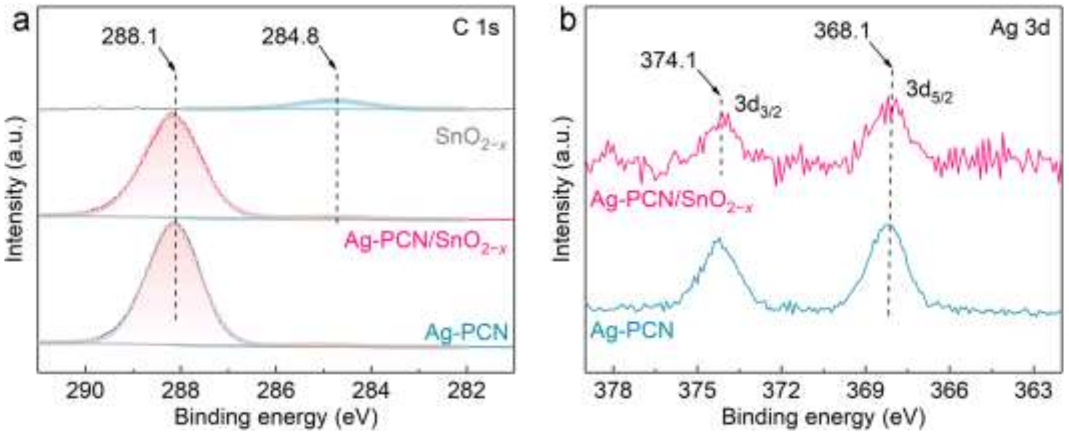

Figure S8. High-resolution (a) C 1s and (b) Ag 3d XPS spectra for Ag-PCN/SnO_{2-x}, Ag-PCN and SnO_{2-x} samples.

As shown in the Ag 3d spectra (Figure S8b), the binding energy of Ag $3d_{2/5}$ at 368.1 eV suggests that Ag is partially positively charged, consistent with the previous report of N-coordinated single Ag atoms.[6]

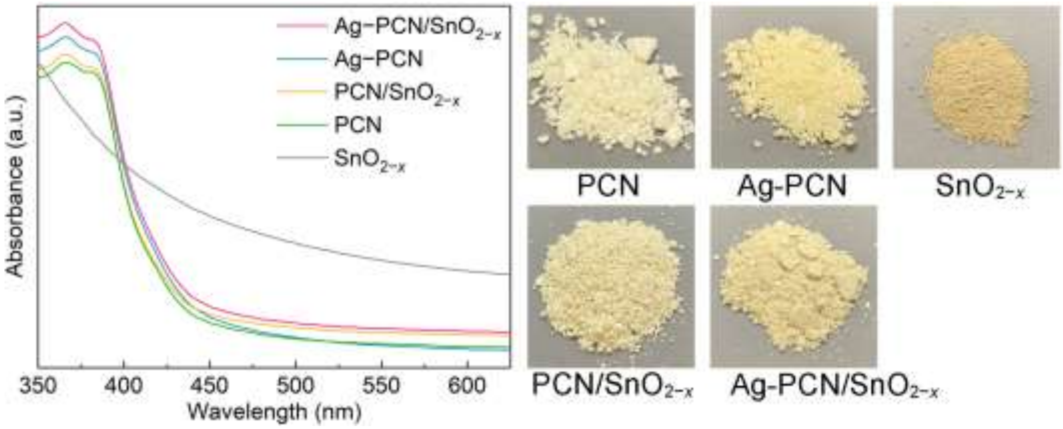

Figure S9. UV-vis DRS spectra and the photographs for Ag-PCN/SnO_{2-x}, Ag-PCN, PCN/SnO_{2-x}, PCN and SnO_{2-x}.

As shown in the UV-vis DRS spectra (Figure S9), both Ag-PCN and SnO_{2-x} exhibit absorption in the visible-light range (λ > 420 nm). The extended light absorption of SnO_{2-x} in the visible-light range corroborates the existence of oxygen vacancies in the sample. No plasmonic absorption band of Ag is observed, consistent with its atomically dispersed state. Compared to PCN, the Ag-PCN displays a slight red shift in the absorption edge, indicating a narrowed bandgap (E_g) after Ag anchoring. The E_g values of the samples are estimated according to the Tauc plots ($(\alpha h\nu)^{1/n}$ versus $h\nu$, where n takes the value of 2 and 1/2 for indirect and direct bandgap semiconductors, respectively),[7] and the results are shown in Figure 2d.

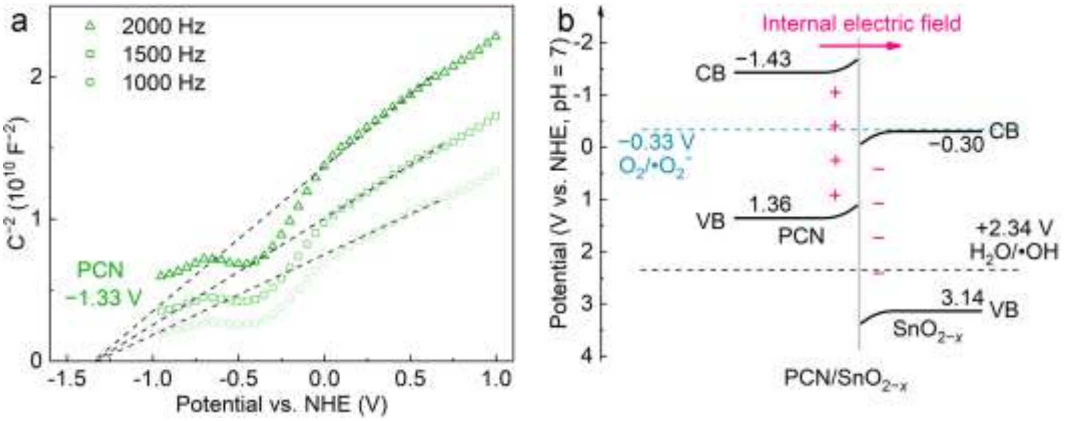

Figure S10. (a) Mott–Schottky plot of PCN. (b) Schematic illustration for the energy band structure of PCN/SnO_{2-x}, highlighting the formation of an internal electric field.

In the PCN/SnO_{2-x} heterojunction, electrons are transferred from PCN to SnO_{2-x}, leading to the formation of an internal electric field pointing from PCN to SnO_{2-x}.[8]

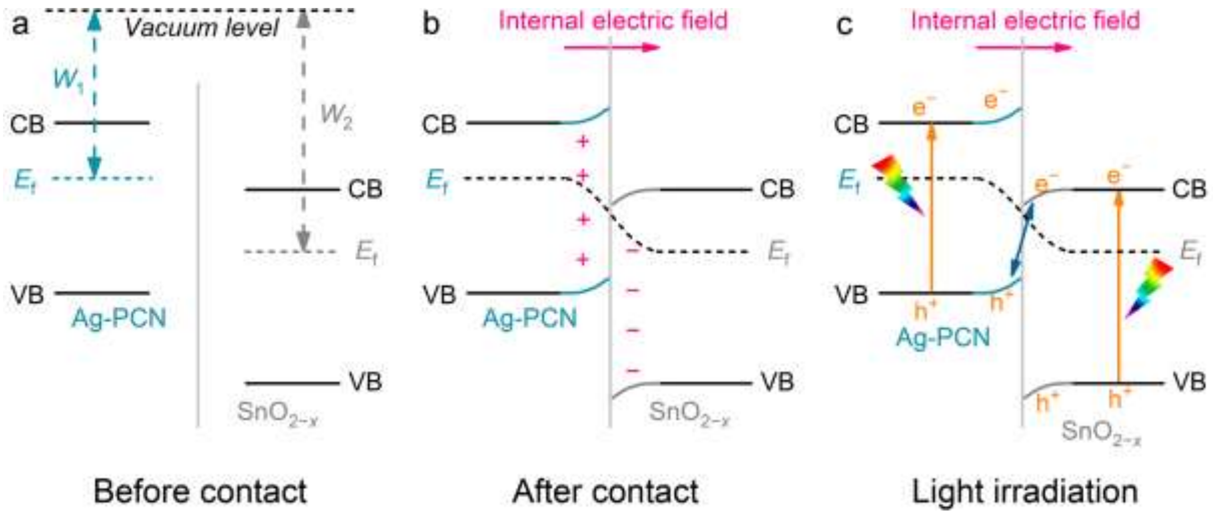

Figure S11. Schematic illustration for the charge transfer process in Ag-PCN/SnO_{2-x}: (a) before contact; (b) after contact; and (c) under light irradiation.

As shown in Figure S11, the Z-scheme heterojunction consists of a reduction photocatalyst (Ag-PCN) and an oxidation photocatalyst (SnO_{2-x}), both of which are n-type semiconductors. Since the Fermi levels of n-type semiconductors are close to their conduction band minimum,[8b] the Ag-PCN (similar to PCN) has a higher Fermi level than SnO_{2-x}. When the two photocatalysts are in contact, electrons in the Ag-PCN will drift to the SnO_{2-x} to balance the Fermi level at the interface. Consequently, the Ag-PCN and SnO_{2-x} close to the interfacial region is positively and negatively charged, respectively, leading to the formation of an internal electric field pointing from Ag-PCN to SnO_{2-x}. Under light irradiation, the internal electric field can assist in the electron transfer from the conduction band of SnO_{2-x} to the valence band of Ag-PCN. In other words, weaker electrons and holes are recombined, while energetic electrons and holes are reserved in the conduction band of Ag-PCN and the valence band of SnO_{2-x}, respectively. As such, electrons and holes can be efficiently separated within the Z-scheme heterojunction, whilst retaining superior redox capabilities for surface catalysis and molecule activation.[8a]

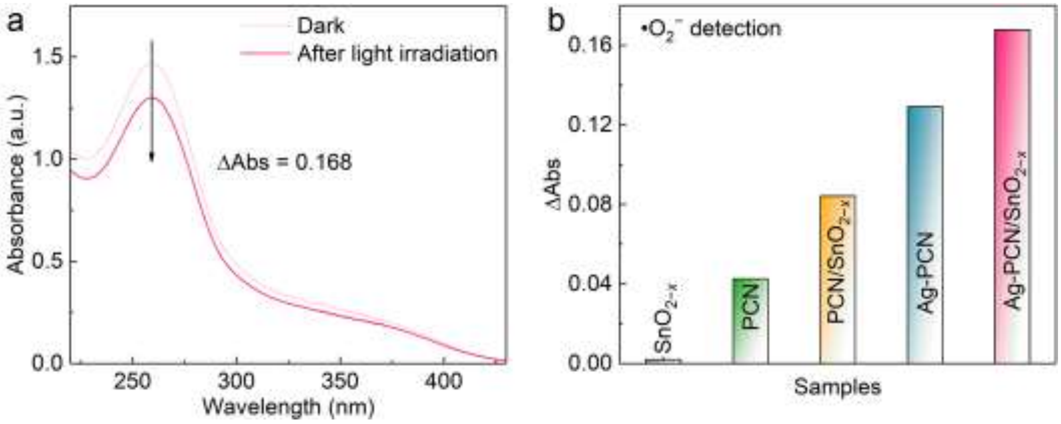

Figure S12. (a) UV-vis absorption spectra of NBT measured in the dark and after 10 min of visible-light irradiation using Ag-PCN/SnO_{2-x} as the catalyst. The characteristic absorption peak of NBT locates at 259 nm. (b) The decrease in the absorbance of NBT after 10 min of visible light irradiation using SnO_{2-x}, PCN, PCN/SnO_{2-x}, Ag-PCN and Ag-PCN/SnO_{2-x} as the catalysts.

To quantify the $•O_2^-$ production over different samples, NBT is employed as an indicator. The reaction between NBT and $•O_2^-$ results in a decrease in the characteristic absorption peak of NBT at 259 nm (Figure S12).[9] Based on the decrease in the NBT absorbance, the $•O_2^-$ production for Ag-PCN/SnO_{2-x} is 3.9- and 2.0-fold that of PCN and PCN/SnO_{2-x}, respectively. No obvious $•O_2^-$ production is observed for SnO_{2-x} due to the weak reduction capability of its photogenerated electrons. These results are in agreement with the ESR observations (Figure 3a).

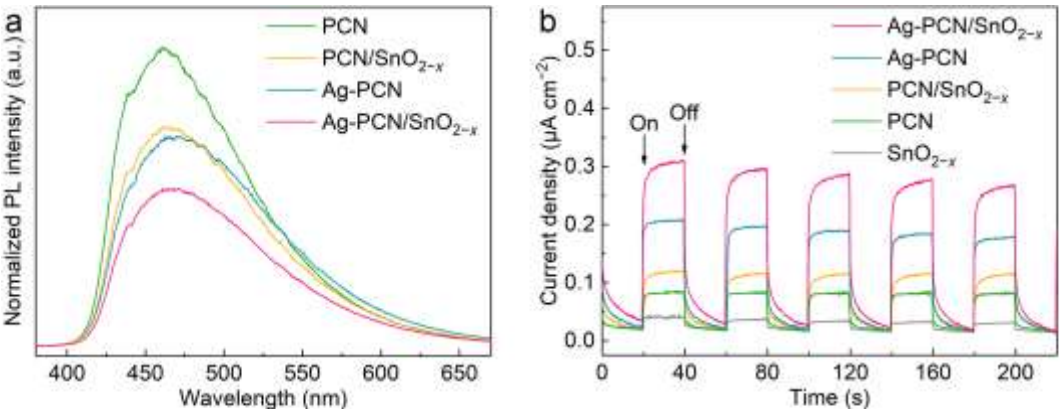

Figure S13. (a) Steady-state PL spectra of PCN, PCN/SnO_{2-x}, Ag-PCN and Ag-PCN/SnO_{2-x} samples. (b) Transient photocurrent responses of SnO_{2-x}, PCN, PCN/SnO_{2-x}, Ag-PCN and Ag-PCN/SnO_{2-x} samples. Transient photocurrent responses are measured at 0.5 V vs. Ag/AgCl in an aqueous solution of 0.2 M Na_2SO_4. The solution is pre-purged with Ar for 15 min before each measurement.

As shown in Figure S13a, pure PCN nanosheets exhibit a PL emission band centered at ca. 460 nm. After either Ag anchoring or SnO_{2-x} loading, the PL intensity of PCN is lessened to some extent. By extension, the simultaneous introduction of Ag and SnO_{2-x} results in the most diminished PL intensity, indicating the Ag and SnO_{2-x} co-promoted charge separation on PCN. This is further corroborated by the highest photocurrent density of Ag-PCN/SnO_{2-x} among all the tested samples (Figure S13b).

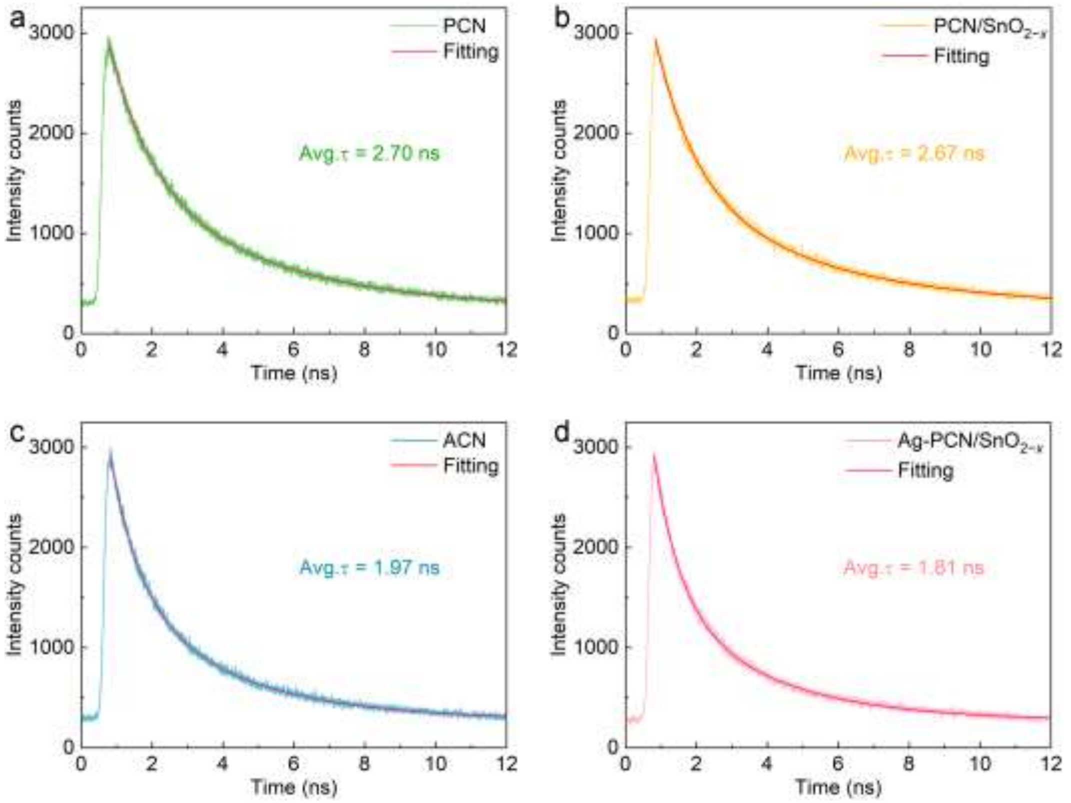

Figure S14. TRPL spectra and the corresponding fitting analysis for (a) PCN, (b) PCN/SnO_{2-x}, (c) Ag-PCN and (d) Ag-PCN/SnO_{2-x} samples. The PL decay profiles are fitted with a bi-exponential model.

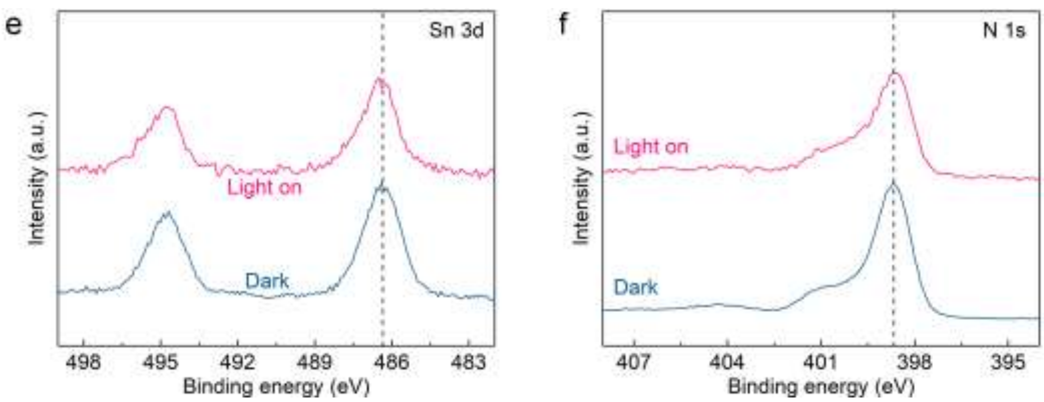

Figure S15. High-resolution (a) Sn 3d and (b) N 1s XPS spectra measured in the dark and under light irradiation for Ag-PCN/SnO_{2-x}.

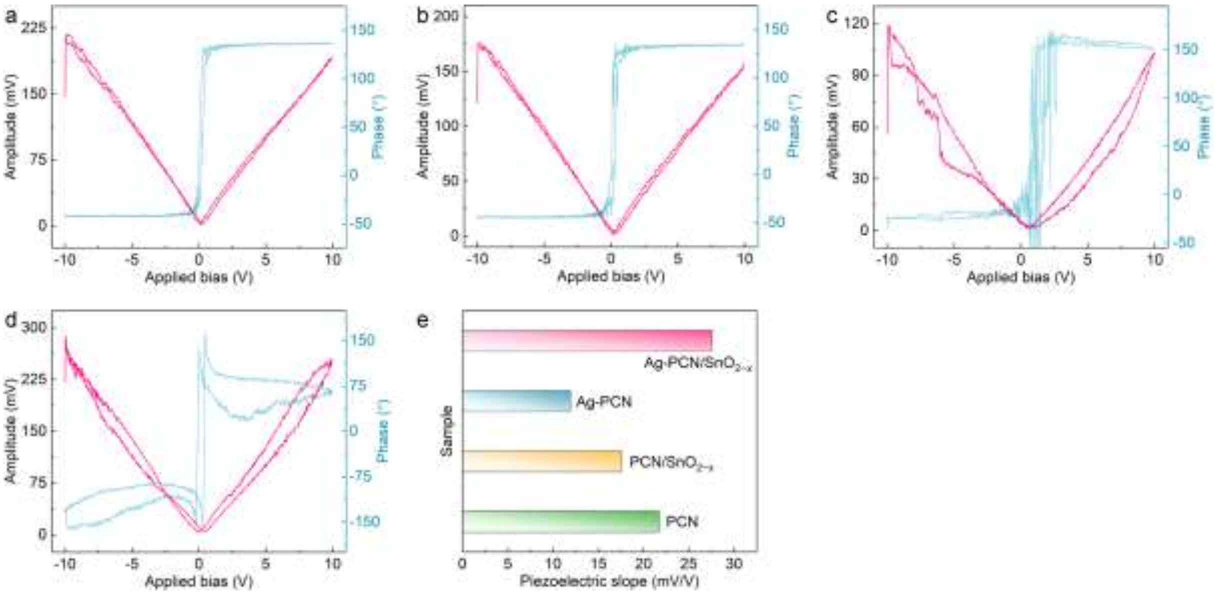

Figure S16. The PFM amplitude–voltage butterfly loops and phase curves for (a) PCN, (b) PCN/SnO_{2-x}, (c) Ag-PCN and (d) Ag-PCN/SnO_{2-x}. (e) The deduced piezoelectric slopes for different samples.

As shown in Figure S16, the amplitude–voltage butterfly loops and the ~180° piezoresponse phase-reversal hysteresis loops verify the piezoelectric feature of PCN-based samples. Since the piezoelectric effect of PCN originates from the superimposed polar tri-s-triazine units and the noncentrosymmetric triangular nanopores,[10] both in-plane (Ag) and out-of-plane (SnO_{2-x}) modifications have the possibility to modify the local asymmetry and piezoresponse of the material.[11] The results show that both Ag and SnO_{2-x} modifications weaken the piezoresponse of PCN. However, compared with PCN (21.7 mV V^{-1}), the Ag-PCN/SnO_{2-x} show a larger piezoelectric slope of 27.6 mV V^{-1}, indicating its stronger piezoelectricity. The single Ag atom anchored in the cavity of PCN shows a similar distance of 2.40 Å to the six neighboring N atoms (Figure 5c), which can lead to a reduced unit-cell dipole moment.[11b] In addition, the electron transfer from PCN to SnO_{2-x} upon the heterojunction formation results in reduced free charge density on PCN. In the case of Ag-PCN/SnO_{2-x}, the existence of Ag accelerates electron transfer between Ag-PCN and SnO_{2-x} under ultrasound irradiation, which potentially makes better utilization of the large quantities of free electrons brought about by oxygen vacancies in SnO_{2-x}. Beyond that, the simultaneous in-plane and out-of-plane charge redistribution can be beneficial for the formation of localized polarization states, thus contributing to the enhanced piezoresponse.[12]

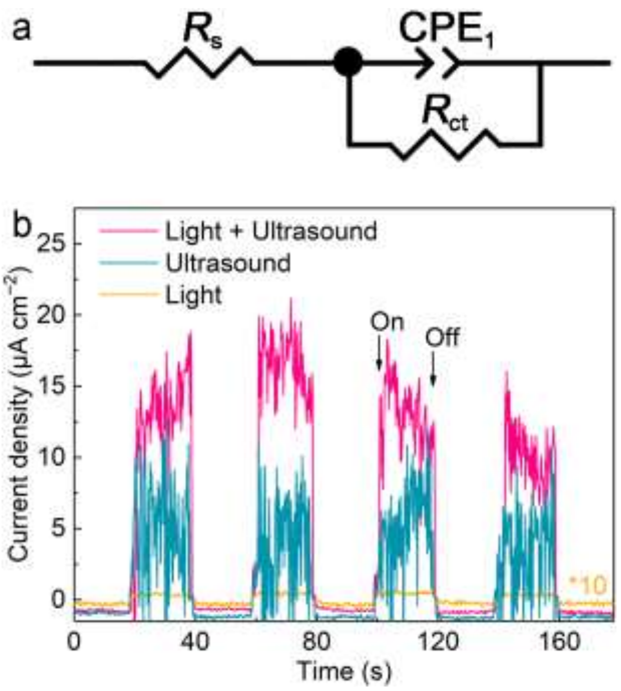

Figure S17. (a) The equivalent electrical circuit used for the fitting analysis of EIS Nyquist plots. In the equivalent circuit, R_s, R_{ct} and CPE_1 represent ohmic series resistance, charge transfer resistance and chemical capacitance at the electrode/electrolyte interface. (b) Transient current responses of Ag-PCN/SnO_{2-x} under different conditions. Data are measured at 0.5 V vs. Ag/AgCl in an aqueous solution of 0.2 M Na_2SO_4. The solution is pre-purged with Ar for 15 min before each measurement. The catalyst electrodes are prepared using carbon cloth as the conductive substrate.

Based on EIS Nyquist plots and the corresponding fitting analysis (Figure 3g and Figure S17a), the charge transfer resistance (R_{ct}) for Ag-PCN/SnO_{2-x} is estimated to be 534.0 kΩ cm^2. The material shows the lowest R_{ct} value (57.2 kΩ cm^2) under the co-irradiation of light and ultrasound. As shown in Figure S17b, the Ag-PCN/SnO_{2-x} exhibits enhanced photoelectric responses under the assistance of ultrasound irradiation. This result suggests that the material photoelectricity and piezoelectricity can collectively promote charge transfer/separation for efficient piezo-photocatalysis.

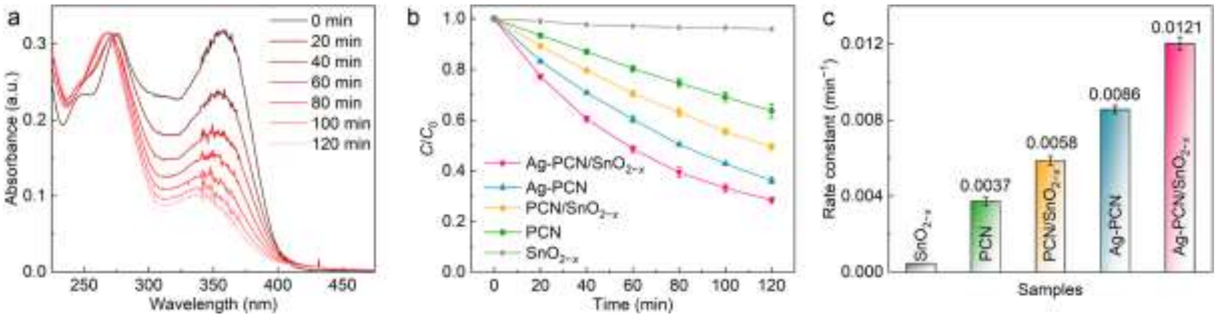

Figure S18. (a) Time-dependent UV-vis absorption spectra collected during photocatalytic TCH degradation using Ag-PCN/SnO_{2-x} as the catalyst. (b) Photocatalytic activities and (c) the deduced rate constants for TCH degradation over Ag-PCN/SnO_{2-x}, Ag-PCN, PCN/SnO_{2-x}, PCN and SnO_{2-x} samples. The error bars in (b) and (c) represent the standard deviation of three independent experiments.

Figure S18a shows the UV-vis results for photocatalytic TCH degradation over Ag-PCN/SnO_{2-x}. The characteristic absorption peak of TCH at 357 nm gradually declines with time, indicating the reduced TCH concentration (Figure S18b). Based on the kinetic plots shown in Figure 4a, the degradation rate constants can be determined. As displayed in Figure S18c, the rate constant for TCH degradation over Ag-PCN/SnO_{2-x} is 0.0121 min^{-1}, 3.3- and 2.1-fold that of PCN (0.0037 min^{-1}) and PCN/SnO_{2-x} (0.0058 min^{-1}), respectively.

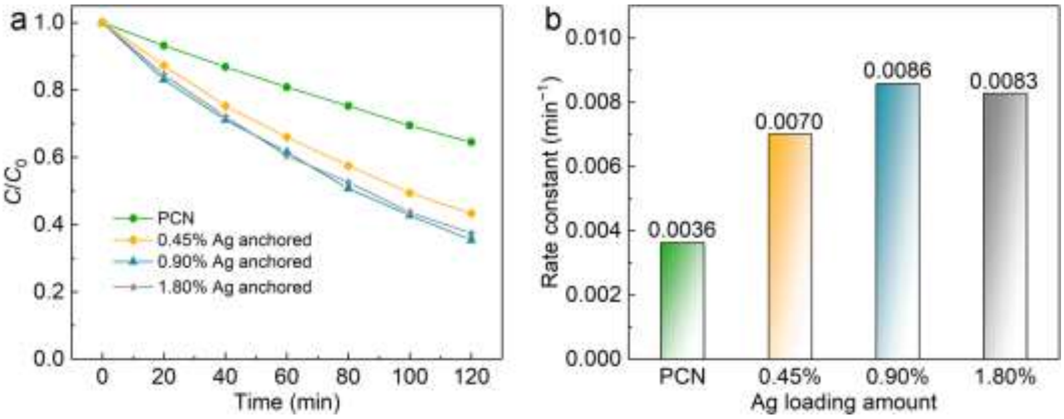

Figure S19. (a) Photocatalytic activities and (b) the deduced rate constants for TCH degradation over Ag-PCN catalysts with different Ag loading amounts.

The Ag loading amount in the Ag-PCN sample is 0.90 wt.% (weight percentage). As shown in Figure S19, further increasing the Ag loading amount to 1.8 wt.% can not enhance the catalytic performance.

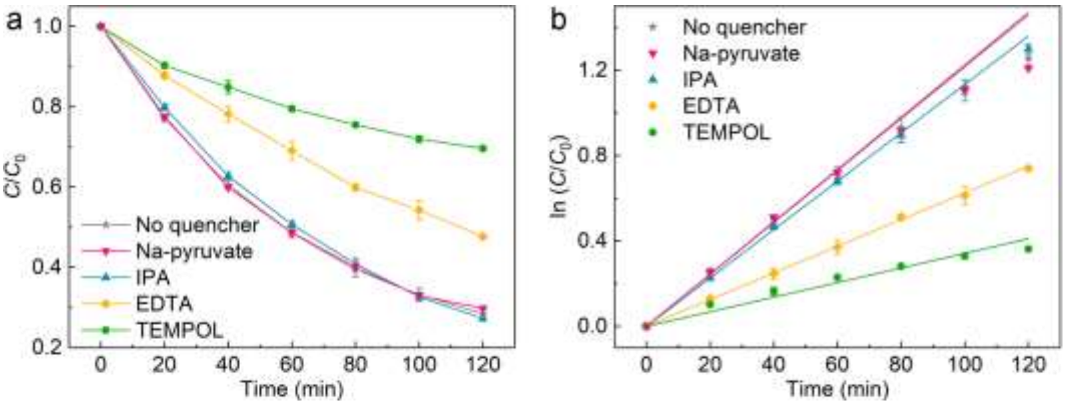

Figure S20. (a) Photocatalytic activities and (b) the corresponding kinetic studies for TCH degradation over Ag-PCN/SnO_{2-x} in the presence of different quenchers, including Na-pyruvate (H_2O_2), IPA (•OH), EDTA (h^+) and TEMPOL ($•O_2^-$). The error bars represent the standard deviation of three independent experiments.

To verify the contribution of $•O_2^-$ in photocatalytic TCH degradation, catalytic experiments are performed over Ag-PCN/SnO_{2-x} in the presence of different quenchers, including Na-pyruvate (H_2O_2), IPA (•OH), EDTA (h^+) and TEMPOL ($•O_2^-$).[13] As shown in Figure S20, the addition of TEMPOL results in the most significant decrease in catalytic performance. Moreover, the performance is dramatically reduced in the absence of O_2 (Figure 4b), corroborating the $•O_2^-$-dominated photocatalytic TCH degradation process.

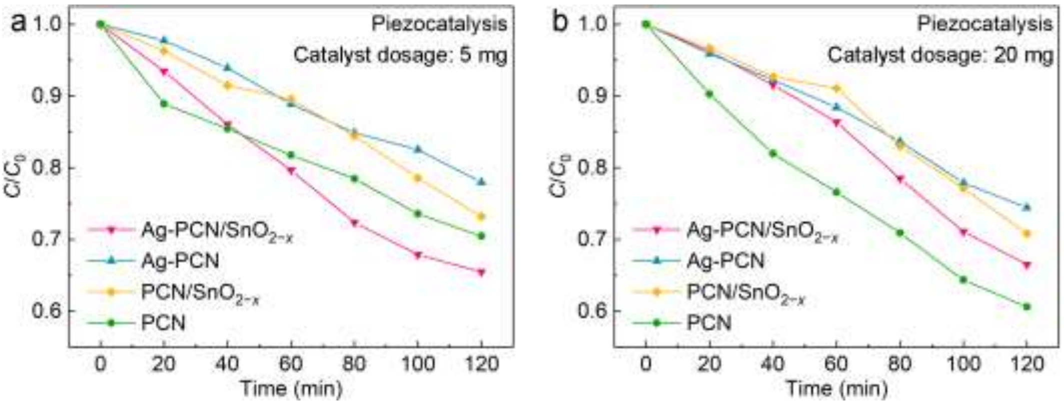

Figure S21. Piezocatalytic activities for TCH degradation using (a) 5 mg and (b) 20 mg of Ag-PCN/SnO_{2-x}, Ag-PCN, PCN/SnO_{2-x} and PCN as the catalysts.

As shown in Figure S21, the piezocatalytic performance of Ag-PCN/SnO_{2-x} outperforms the other samples at a catalyst dosage of 5 mg, consistent with its strongest piezoresponse. Generally, increasing the catalyst dosage can produce more charge carriers for piezocatalysis. However, the performance of Ag-PCN/SnO_{2-x} is slightly reduced at high catalyst dosages (20 mg). The reduced piezocatalytic performance at high catalyst dosages can be explained as the increased collision probability between catalysts leads to the quenching effect between positive and negative charges.[14]

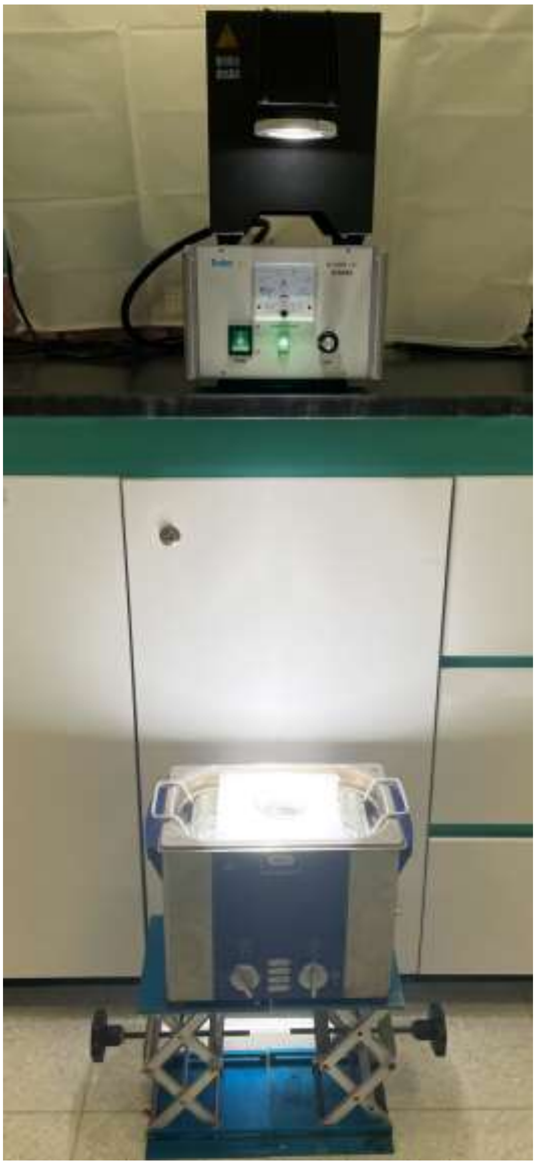

Figure S22. Photograph of the reaction setup for piezo-photocatalysis.

As shown in Figure S22, the visible light ($\lambda > 420$ nm) and ultrasound (37 kHZ) irradiation were provided by a 300 W xenon lamp (Perfect Light, PLS 300+) and a 280 W ultrasonicator (Elmasonic S 30H), respectively.

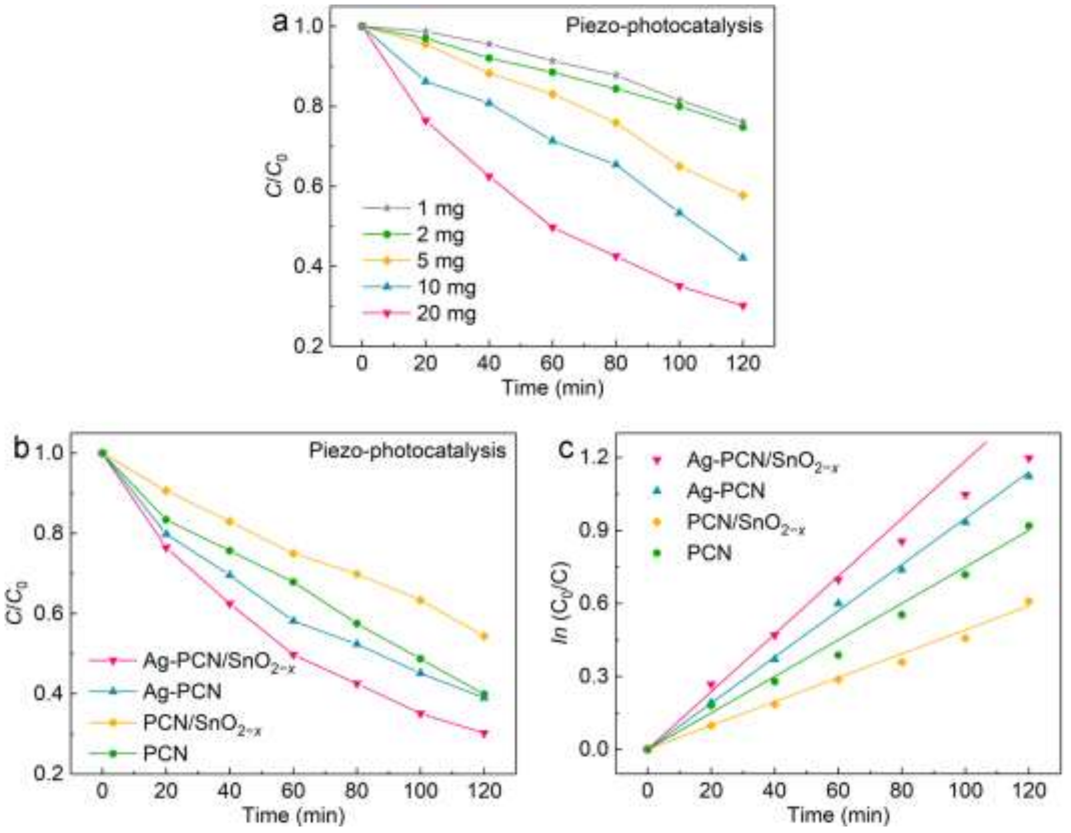

Figure S23. (a) Piezo-photocatalytic activities for TCH degradation over Ag-PCN/SnO_{2-x} at different catalyst dosages. (b) Piezo-photocatalytic activities and (c) the corresponding kinetic studies for TCH degradation over Ag-PCN/SnO_{2-x}, Ag-PCN, PCN/SnO_{2-x} and PCN.

Although the piezocatalytic performance of Ag-PCN/SnO_{2-x} reaches the maximum at a catalyst dosage of 10 mg (Figure 4c), the piezo-photocatalytic performance of the material increases with increasing catalyst dosages (Figure S23a). This result emphasizes the importance of light energy harvesting in piezo-photocatalysis. The superior photo- and piezo-responses of the single Ag atoms-integrated Z-scheme heterojunction (i.e., Ag-PCN/SnO_{2-x}) bestow it with the highest piezo-photocatalytic performance among all the tested samples (Figure S23b,c). The apparent rate constant of piezo-photocatalytic degradation reaches 0.0116 min^{-1} for Ag-PCN/SnO_{2-x}, 1.5 and 2.4 times that of PCN and PCN/SnO_{2-x}, respectively.

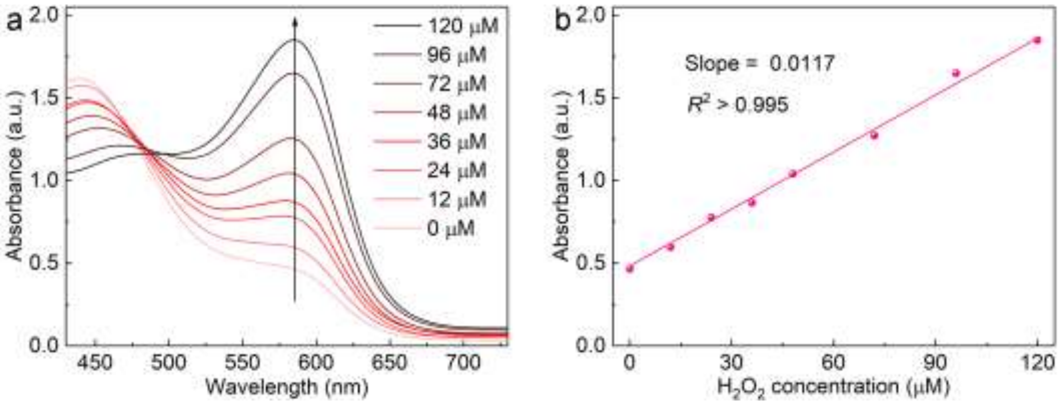

Figure S24. (a) Determination of H_2O_2 production over Ag-PCN/SnO_{2-x} using colorimetric assay kits (MAK311-1KT, Sigma-Aldrich). (b) The characteristic absorption of the Fe^{3+}–xylenol orange complex at 585 nm as a function of the H_2O_2 concentration.

The H_2O_2 production is quantitatively determined using a commercial colorimetric assay kit (MAK311-1KT, Sigma-Aldrich). This assay kit leverages a chromogenic reaction in which the oxidation of Fe^{2+} to Fe^{3+} by peroxides generates a purple complex (Fe^{3+}–xylenol orange) in the presence of xylenol orange. As shown in Figure S24, the absorbance of the generated complex at 585 nm is proportional to the H_2O_2 concentration.

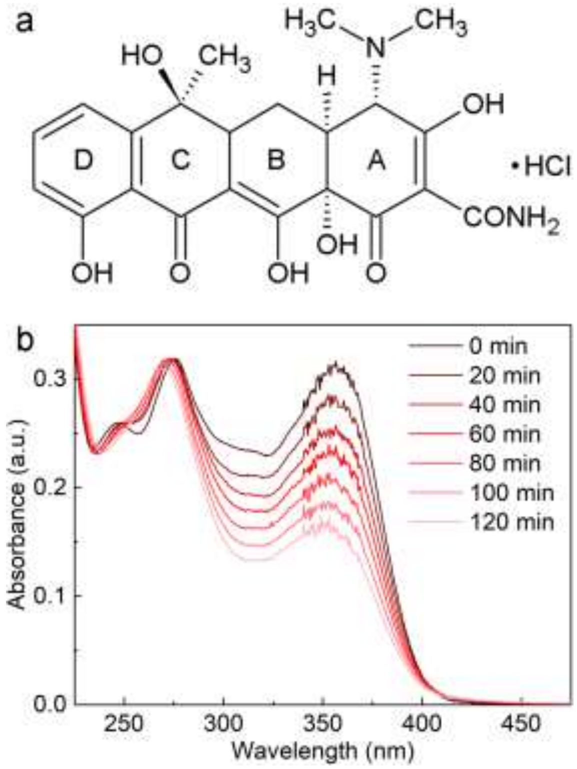

Figure S25. (a) Chemical structure of TCH. (b) Time-dependent UV-vis spectra for piezo-photocatalytic TCH degradation over Ag-PCN/SnO_{2-x} in the presence of IPA.

In the UV-vis spectra, TCH shows two major absorption bands at around 275 and 357 nm. The peak at 357 nm is associated with the aromatic rings B, C and D including the extended chromophores.[15] Meanwhile, the peak at 275 nm originates from the aromatic ring A including the amide, ketone and enolic hydroxyl groups. The reduced peak intensity at 357 nm can be attributed to the destruction of the aromatic rings B, C and D under the attack of ROS. In addition, the decay of the peak at 275 nm is considered to stem from the degradation of amide and enolic groups connected to the aromatic ring A in TCH and its degradation intermediates.[16] In photocatalytic TCH degradation, the peak at 357 nm gradually decays with time. However, the intensity for the peak at 275 nm remains almost unchanged, indicating the formation of refractory TCH degradation intermediates. Interestingly, in the piezo-photocatalytic TCH degradation process, the refractory intermediates can be further degraded, as evidenced by the gradually reduced peak intensity at 275 nm (Figure 4e). Based on the scavenger experiments (Figure 4f), this result can be understood by the promoted •OH production during the piezo-photocatalytic process. It is also worth mentioning that when IPA is introduced into the piezo-photocatalytic system to quench the •OH radicals, the peak intensity at 275 nm of TCH remains almost unchanged during the reaction (Figure S25b). This result further highlights the role of •OH radicals in promoting the deep removal of TCH.

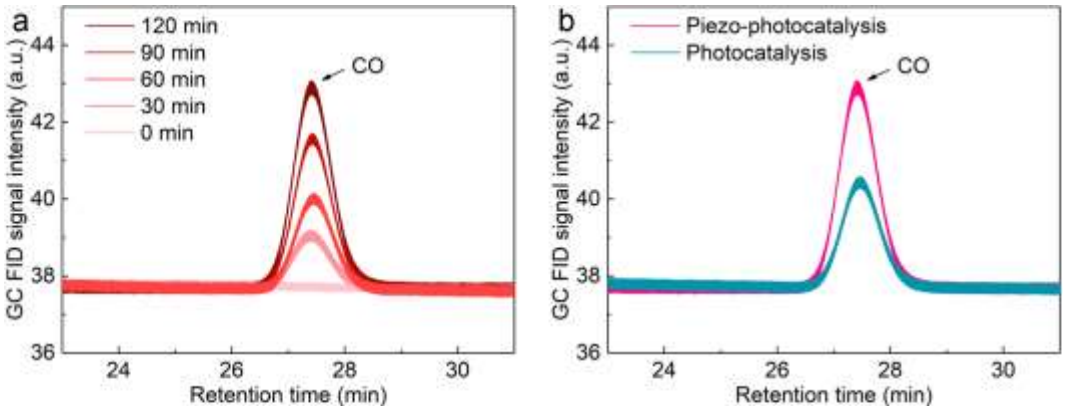

Figure S26. (a) GC detection of the CO produced during piezo-photocatalytic TCH degradation. (b) Comparison of CO production between piezo-photocatalysis and photocatalysis after 120 min of reaction.

To provide more insight into the photocatalytic and piezo-photocatalytic degradation process, we employ gas chromatography (GC) to detect the gaseous products of the reaction (mainly CO, the CO_2 amount in the system can not be accurately determined). As shown in the newly added Figure S26, the CO product gradually accumulates in the piezo-photocatalytic system. Moreover, compared to photocatalysis, the piezo-photocatalysis shows enhanced CO production, corroborating the piezo-assisted deep removal of refractory pollutants.

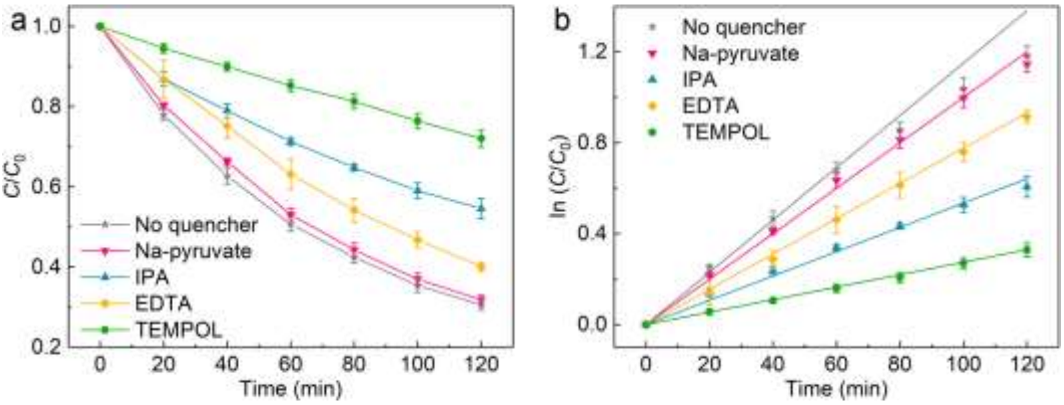

Figure S27. (a) Piezo-photocatalytic activities and (b) the corresponding kinetic studies for TCH degradation over Ag-PCN/SnO_{2-x} in the presence of different quenchers, including Na-pyruvate (H_2O_2), IPA (•OH), EDTA (h^+) and TEMPOL (•O_2^-). The error bars in (a) and (b) represent the standard deviation of three independent experiments.

As shown in Figure S27, •OH radicals play an important role (53.1%) in piezo-photocatalysis. •OH radicals possess higher oxidation capability than •O_2^- radicals, successfully explaining the piezo-assisted deep removal of TCH.

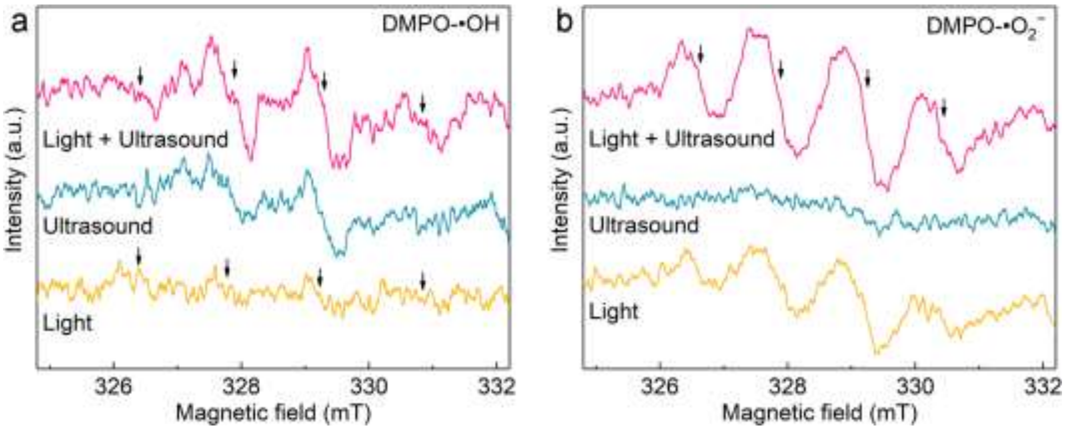

Figure S28. ESR spectra of (a) •OH and (b) $•O_2^-$ captured under different conditions using Ag-PCN/SnO_{2-x} as the catalyst and DMPO as a spin-trapping agent.

As shown in the newly added Figure S28, the characteristic ESR signals of DMPO-•OH and DMPO-$•O_2^-$ are observed for Ag-PCN/SnO_{2-x} under the co-irradiation of light and ultrasound. Moreover, the significantly enhanced signal intensity for both DMPO-•OH and DMPO-$•O_2^-$ in piezo-photocatalysis suggests the synergy of light and ultrasound in promoting molecule activation and ROS production.

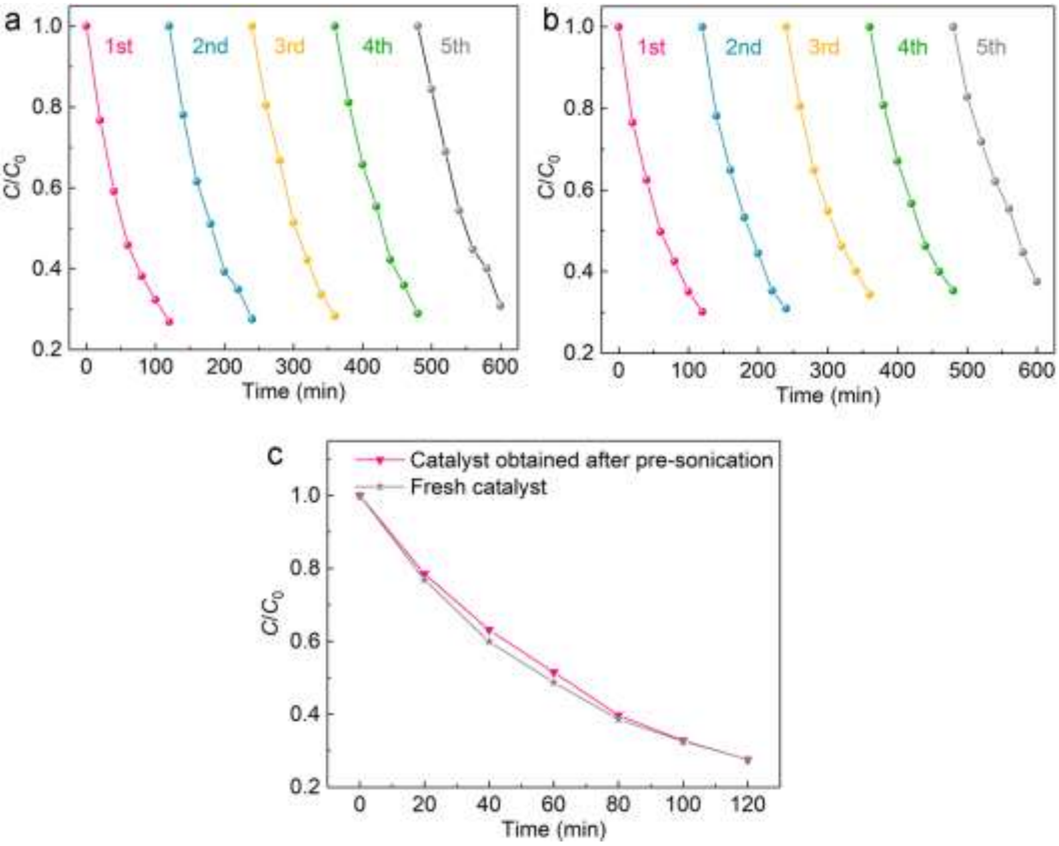

Figure S29. Cyclic tests for (a) photocatalytic and (b) piezo-photocatalytic TCH degradation using Ag-PCN/SnO_{2-x} as the catalyst. (c) Photocatalytic performance of the Ag-PCN/SnO_{2-x} catalyst obtained after 60 min of pre-sonication.

As shown in the updated Figure S29a,b, the catalytic performance of the Ag-PCN/SnO_{2-x} catalyst has no significant change during five consecutive tests. In addition, we have examined the photocatalytic performance of the catalyst obtained after 60 min of pre-sonication. As shown in Figure S29c, the pre-sonication treatment has no obvious influence on the photocatalytic performance of Ag-PCN/SnO_{2-x}. This result indicates that the surface status of the catalyst is well maintained under ultrasound irradiation.

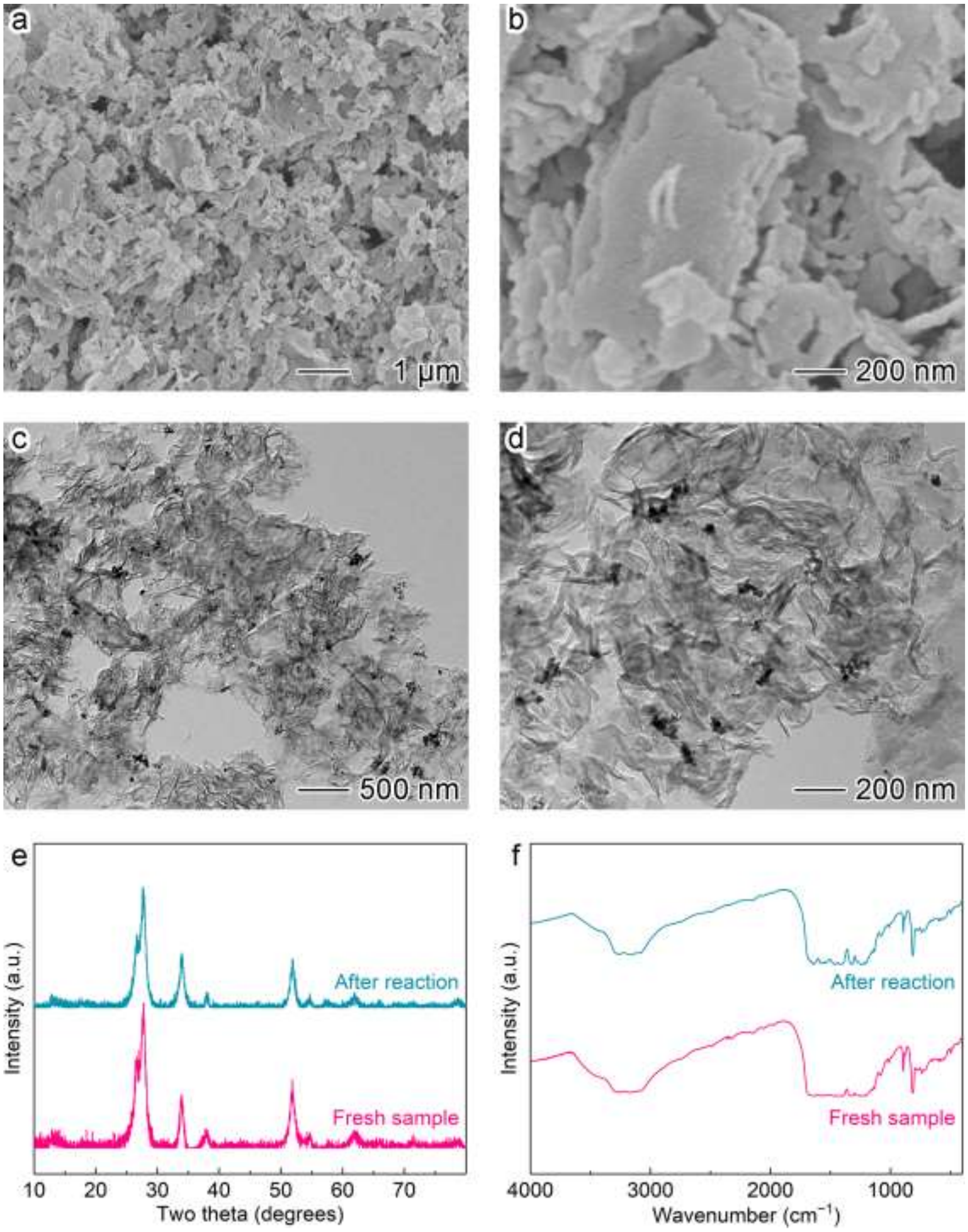

Figure S30. (a,b) SEM images, (c,d) TEM images, (e) XRD pattern and (f) FTIR spectra of the Ag-PCN/SnO_{2-x} sample collected after cyclic tests.

As shown in Figure S30, the morphology and structure of the Ag-PCN/SnO_{2-x} catalyst are well maintained after cyclic tests.

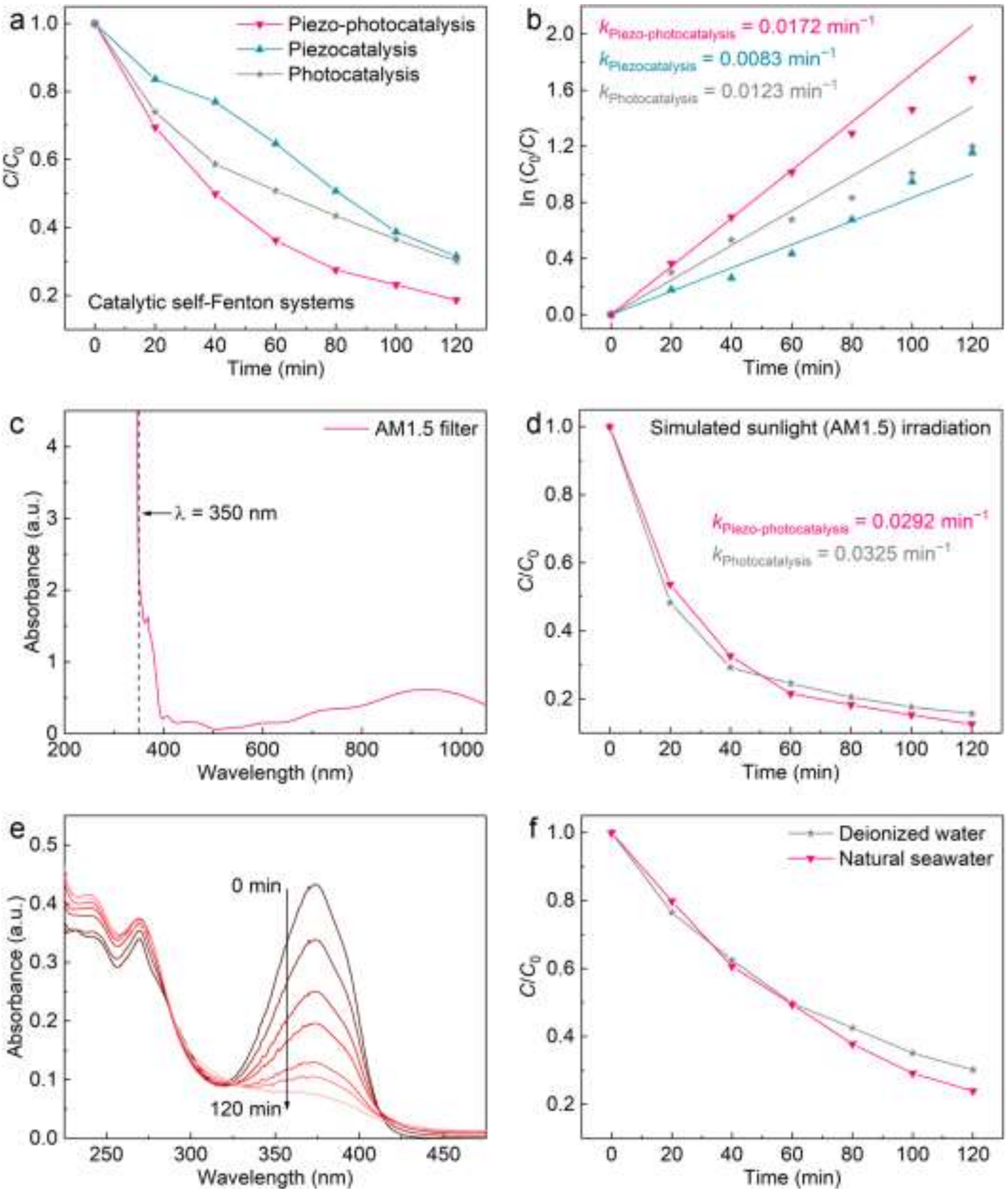

Figure S31. (a) Catalytic activities for TCH degradation over Ag-PCN/SnO_{2-x} in the presence of Fe^{3+} (catalytic self-Fenton systems) and (b) the corresponding kinetic analysis. (c) Absorption spectra of the AM1.5 light filter used for obtaining simulated sunlight. (d) Photocatalytic and piezo-photocatalytic activities for TCH degradation over Ag-PCN/SnO_{2-x} under simulated sunlight irradiation. (e) Time-dependent UV-vis spectra for piezo-photocatalytic TCH degradation in natural seawater using Ag-PCN/SnO_{2-x} as the catalyst. (f) Comparison of piezo-photocatalytic performance measured in natural seawater and deionized water using Ag-PCN/SnO_{2-x} as the catalyst.

By introducing a trace amount of Fe^{3+} (10 μmol L^{-1}) into the reaction mixture to construct a piezo-photocatalytic self-Fenton system, the TCH degradation rate is optimized from 0.0116 to 0.0172 min^{-1} (Figure S31a,b). Noteworthy, the photocatalytic performance only slightly increases to 0.0123 min^{-1} in the presence of Fe^{3+}. The performance of the piezo-

photocatalytic self-Fenton system is 1.4 and 2.1 times that of photocatalytic self-Fenton and piezocatalytic self-Fenton systems, respectively, demonstrating its superiority for efficient ROS production and pollutant degradation. To better test the practicability of Ag-PCN/SnO_{2-x}, simulated sunlight (AM1.5) is used as the light source for TCH degradation. The light intensity is 15.3 mW cm^{-2}, close to the actual light intensity outdoors. As shown in Figure S31d, the rate constant for piezo-photocatalytic TCH degradation is determined to be 0.0292 min^{-1} when simulated sunlight is introduced. This performance is slightly lower than that of photocatalysis powered by simulated sunlight (0.0325 min^{-1}), which can be attributed to reduced collision probability between antibiotic pollutants and catalyst surfaces under ultrasound irradiation.

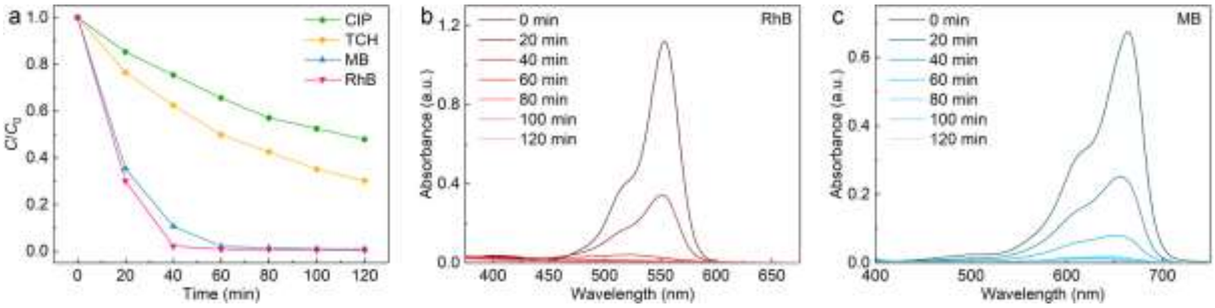

Figure S32. (a) Piezo-photocatalytic degradation of different pollutants using Ag-PCN/SnO_{2-x} as the catalyst. Reaction conditions: 20 mg catalysts, 10 mg L^{-1} pollutants and 1 atm air atmosphere. Time-dependent UV-vis absorption spectra collected during photocatalytic degradation of (b) RhB and (c) MB using Ag-PCN/SnO_{2-x} as the catalyst.

As shown in Figure S32, other organic pollutants, including ciprofloxacin (CIP), rhodamine B (RhB) and methylene blue (MB) can also be efficiently degraded through piezo-photocatalysis using Ag-PCN/SnO_{2-x} as the catalyst. As shown in Figure S32b and Table S2, the Ag-PCN/SnO_{2-x} exhibits a competitive performance with other recently reported piezocatalysts/piezo-photocatalysts. Specifically, the degradation rate of RhB reaches 97.8% within 40 min over the Ag-PCN/SnO_{2-x} catalyst.

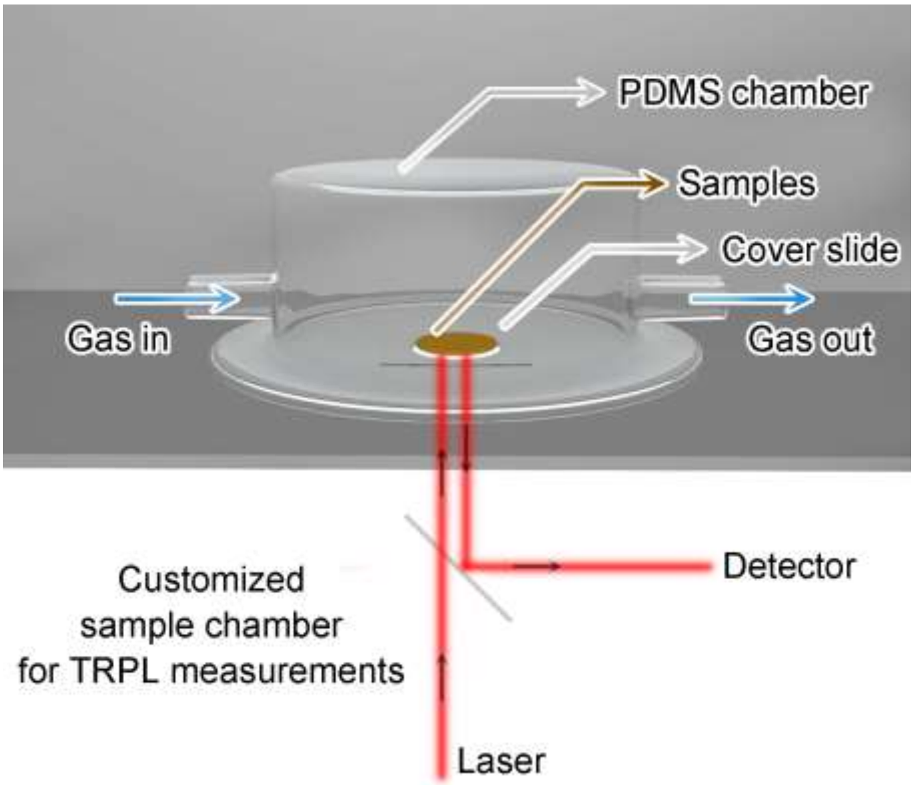

Figure S33. Schematic illustration for the customized sample chamber for TRPL measurements.

Figure S33 shows the customized sample chamber for TRPL measurements. The sample chamber is made of polydimethylsiloxane (PDMS), allowing the system to be light-permittable and gas-tight. By using this chamber, TRPL measurements can be carried out under different atmospheres, including Ar and O_2.

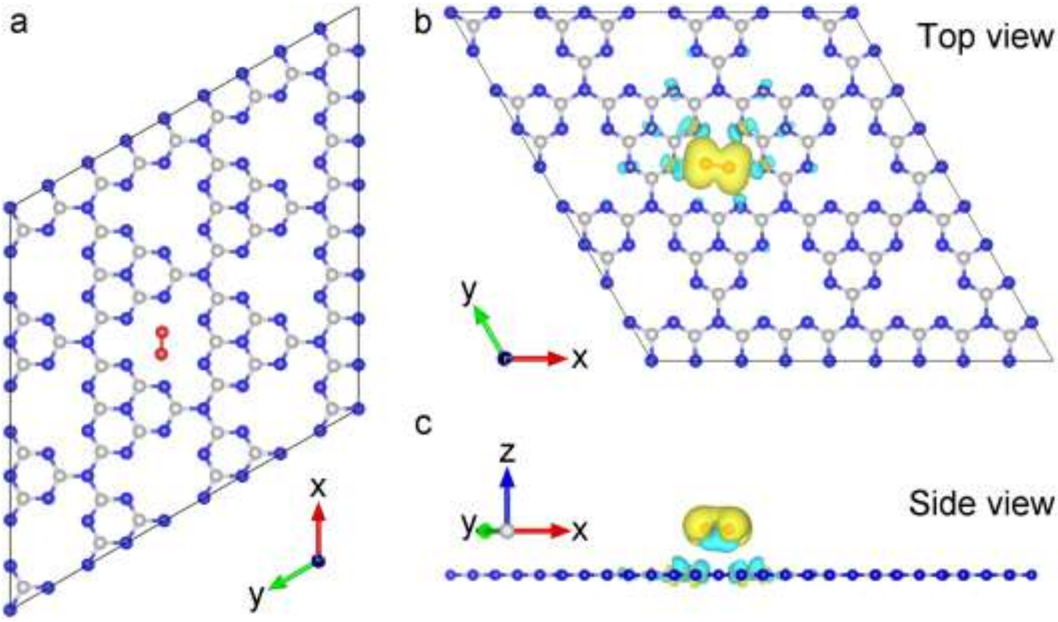

Figure S34. (a) Structure models for O_2 adsorption on PCN nanosheets and (b,c) the corresponding charge density differences. The grey, blue and red balls represent C, N and O atoms, respectively. The yellow and cyan regions represent electron accumulation and electron depletion, respectively.

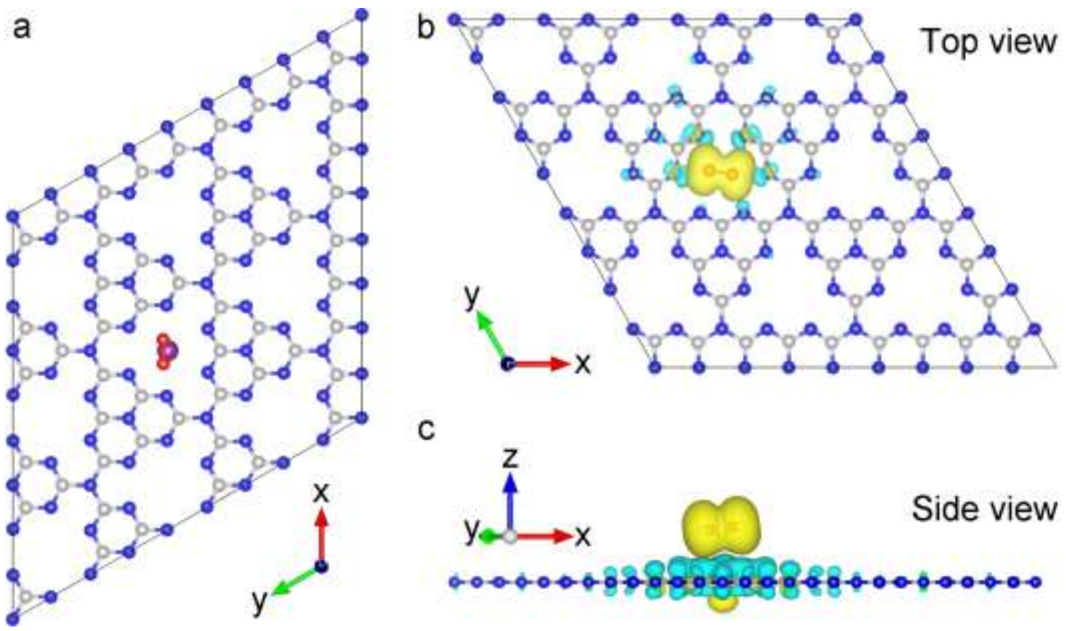

Figure S35. (a) Structure models for O_2 adsorption on Ag-PCN nanosheets and (b,c) the corresponding charge density differences. The grey, blue, purple and red balls represent C, N, Ag and O atoms, respectively. The yellow and cyan regions represent electron accumulation and electron depletion, respectively.

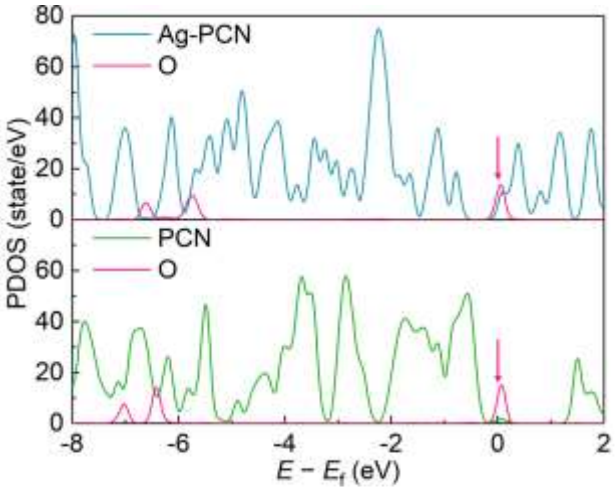

Figure S36. The projected density of states (PDOS) for O_2-adsorbed Ag-PCN (top) and O_2-adsorbed PCN (bottom) systems. The blue, pink and green lines correspond to the PDOS of Ag-PCN, O_2 and PCN, respectively. The red color arrows indicate the orbital overlapping between the substrate and O_2 near the Fermi level.

Figure S36 shows the calculated PDOS for O_2-adsorbed Ag-PCN and O_2-adsorbed PCN systems. Compared with PCN, the Ag-PCN substrate shows enhanced orbital overlapping with the O near the Fermi level. This result indicates the stronger electronic interaction between O_2 and the Ag-containing substrate, which is in line with the calculation results of charge transfer and adsorption energy.

Table S1. PL lifetime and their relative percentages for different samples (λ_{ex} = 374 nm).

Sample	A_1	τ_1 (ns)	A_2	τ_2 (ns)	τ_{ave} (ns)[a]	η
PCN	0.480	1.18	0.520	4.10	2.70	
Ag-PCN	0.516	0.82	0.484	3.20	1.97	26.9%[b]
PCN/SnO_{2-x}	0.530	1.15	0.470	4.38	2.67	
Ag-PCN/SnO_{2-x}	0.539	0.767	0.461	3.022	1.81	32.3%[c]

[a]The average PL lifetime, τ_{ave} is calculated as follows:

$$\tau_{ave}=\sum_{i=0}^{n} A_i \tau_i$$

[b]The electron transfer efficiency from PCN to Ag, η_1 is calculated as follows:

$$\eta_1=\frac{\frac{1}{\tau_{Ag\text{-}PCN}}-\frac{1}{\tau_{PCN}}}{\frac{1}{\tau_{Ag\text{-}PCN}}}=26.9\%$$

[c]The electron transfer efficiency from PCN/SnO_{2-x} to Ag, η_2 is calculated as follows:

$$\eta_2=\frac{\frac{1}{\tau_{Ag\text{-}PCN/SnO2\text{-}x}}-\frac{1}{\tau_{PCN/SnO2\text{-}x}}}{\frac{1}{\tau_{Ag\text{-}PCN/SnO2\text{-}x}}}=32.3\%$$

Table S2. Performance comparison for recently reported systems on piezocatalytic/piezo-photocatalytic pollutant degradation[a)]

Catalysts	Dosages		Degradation rate	Reaction conditions	Ref.
	Catalysts [mg]	RhB [mg L^{-1}]			
Ag-PCN/SnO_{2-x}	20	10	97.8% in 40 min	300 W Xe lamp (> 420 nm), 280 W ultrasonicator (37 kHz)	This work
Au/$AgNbO_3$	50	10	80% in 30 min	300 W Xe lamp (> 420 nm), 110 W ultrasonicator (40 kHz)	[17]
C_3N_{5-x}-O	10	20	95% in 30 min	Ultrasonicator (KQQ-300DE)	[18]
C_3N_{5-x}-CN	10	20	99% in 30 min	300 W Xe lamp (> 420 nm), ultrasonicator (KQQ-300DE)	[19]
$BaTiO_3$ nanobelts	50	10	82% in 70 min	100 W ultrasonicator (50 kHz)	[20]
ZnO/$BaTiO_3$	100	10	97% in 30 min	500 W Xe lamp (300–900 nm), 200 W ultrasonicator (40 kHz)	[21]
$BaTiO_3$/TiO_2	100	10	100% in 60 min	250 W mercury lamp (365 nm), 300 W ultrasonicator (40 kHz)	[22]
$BaTiO_3$@TiO_2	50	5	99.5% in 75 min	300 W Xe lamp (full spectrum), 200 W ultrasonicator (45 kHz)	[23]
$BaTiO_3$@ReS_2	20	10	95.8% in 25 min	Xe lamp (full spectrum), 100 W ultrasonicator (40 kHz)	[11a]
$BaTiO_3$@C	100	10	100% in100 min	300 W Xe lamp (AM 1.5G), 120 W ultrasonicator (40 kHz)	[24]
$Ba(Zr_xTi_{1-x})O_3$	50	5	95% in 90 min	180 W ultrasonicator (40 kHz)	[25]
$Bi_{0.5}Na_{0.5}TiO_3$	10	5	95% in 30 min	300 W Xe lamp (full spectrum), 200 W ultrasonicator (28 kHz)	[26]
$Bi_{0.5}Na_{0.5}TiO_3$ @$BiVO_4$	100	10	93.8% in 60 min	300 W Xe lamp (AM 1.5G), 200 W ultrasonicator (28 kHz)	[27]
BiVO4:I/ $BaTiO_3$-Ag	20	10	55% in 15 min	Xe lamp (>380 nm), 150 W ultrasonicator (40 kHz)	[28]
$Na_{0.5}K_{0.5}NbO_3$-$LiNbO_3$	200	5	91% in 100 min	300 W ultrasonicator (40 kHz)	[29]
Few-layer MoS_2	10	10	96% in 60 min	110 W ultrasonicator (40 kHz)	[30]
MoS_2@TiO_2	50	20	99% in 10 min	Magnetic stirrer (600 r min^{-1})	[31]
MoS_2/rGO	10	10	90% in 20 s	200 W ultrasonicator (40 kHz)	[32]

[a)]RhB is chosen as the model substrate for a better performance comparison.

Table S3. PL lifetime and their relative percentages for different samples under O_2 atmosphere (λ_{ex} = 374 nm).

Sample	A_1	τ_1 (ns)	A_2	τ_2 (ns)	τ_{ave} (ns)[a]
Ag-PCN	0.536	0.78	0.464	2.93	1.78
PCN/SnO_{2-x}	0.534	1.19	0.466	4.42	2.69
Ag-PCN/SnO_{2-x}	0.527	0.67	0.473	2.83	1.69

[a]The average PL lifetime, τ_{ave} is calculated as follows:

$$\tau_{ave} = \sum_{i=0}^{n} A_i \tau_i$$

Table S4. Calculated energies for O_2 adsorption on PCN and Ag-PCN.

Model	Adsorption energy (eV)[a]	O–O bond length (Å)	Electron transfer[b]
PCN + O_2	−0.22	1.24	0.12
Ag-PCN + O_2	−0.67	1.28	0.40

[a]Energy for O_2 adsorption on the surface of the catalyst model.
[b]Electron transfer from the catalyst model to adsorbed O_2.

References

[1] S. Sun, G. Shen, J. Jiang, W. Mi, X. Liu, L. Pan, X. Zhang, J. J. Zou, *Adv. Energy Mater.* **2019**, *9*, 1901505.

[2] X. Li, S. Zhao, X. Duan, H. Zhang, S.-z. Yang, P. Zhang, S. P. Jiang, S. Liu, H. Sun, S. Wang, *Appl. Catal. B Environ.* **2021**, *283*, 119660.

[3] K. Li, L. Wang, Z. Chen, X. Yang, Y. X. Yu, W. D. Zhang, Y. Wang, Y. Shi, K. P. Loh, Q. H. Xu, *Adv. Funct. Mater.* **2020**, *30*, 2005106.

[4] J. Jiang, D. Duan, J. Ma, Y. Jiang, R. Long, C. Gao, Y. Xiong, *Appl. Catal. B Environ.* **2021**, *295*, 120261.

[5] Y. He, L. Zhang, M. Fan, X. Wang, M. L. Walbridge, Q. Nong, Y. Wu, L. Zhao, *Sol. Energy Mater. Sol. Cells* **2015**, *137*, 175.

[6] S. Tian, C. Peng, J. Dong, Q. Xu, Z. Chen, D. Zhai, Y. Wang, L. Gu, P. Hu, H. Duan, D. Wang, Y. Li, *ACS Catal.* **2021**, *11*, 4946.

[7] J. Wang, L. Xu, T. Wang, R. Li, Y. Zhang, J. Zhang, T. Peng, *Adv. Energy Mater.* **2021**, *11*, 2003575.

[8] a) L. Zhang, J. Zhang, H. Yu, J. Yu, *Adv. Mater.* **2021**, *33*, e2107668; b) P. Xia, S. Cao, B. Zhu, M. Liu, M. Shi, J. Yu, Y. Zhang, *Angew. Chem., Int. Ed.* **2020**, *59*, 5218.

[9] D. Wang, H. Wang, L. Ji, M. Xu, B. Bai, X. Wan, D. Hou, Z. Y. Qiao, H. Wang, J. Zhang, *ACS Nano* **2021**, *15*, 8694.

[10] C. Hu, F. Chen, Y. Wang, N. Tian, T. Ma, Y. Zhang, H. Huang, *Adv. Mater.* **2021**, *33*, e2101751.

[11] a) W. Liu, P. Wang, Y. Ao, J. Chen, X. Gao, B. Jia, T. Ma, *Adv. Mater.* **2022**, *34*, e2202508; b) C. Hu, J. Hu, Z. Zhu, Y. Lu, S. Chu, T. Ma, Y. Zhang, H. Huang, *Angew. Chem., Int. Ed.* **2022**, *61*, e202212397.

[12] C. Wang, C. Hu, F. Chen, T. Ma, Y. Zhang, H. Huang, *Nano Energy* **2023**, *107*, 108093.

[13] a) X. Chu, Y. Qu, A. Zada, L. Bai, Z. Li, F. Yang, L. Zhao, G. Zhang, X. Sun, Z. D. Yang, L. Jing, *Adv. Sci.* **2020**, *7*, 2001543; b) Y. Deng, J. Liu, Y. Huang, M. Ma, K. Liu, X. Dou, Z. Wang, S. Qu, Z. Wang, *Adv. Funct. Mater.* **2020**, *30*, 2002353.

[14] a) J. Ma, S. Jing, Y. Wang, X. Liu, L. Y. Gan, C. Wang, J. Y. Dai, X. Han, X. Zhou, *Adv. Energy Mater.* **2022**, *12*, 2200253; b) R. Su, H. A. Hsain, M. Wu, D. Zhang, X. Hu, Z. Wang, X. Wang, F. t. Li, X. Chen, L. Zhu, *Angew. Chem., Int. Ed.* **2019**, *58*, 15076.

[15] H. Wang, T. Chen, D. Chen, X. Zou, M. Li, F. Huang, F. Sun, C. Wang, D. Shu, H. Liu, *Appl. Catal. B Environ.* **2020**, *260*, 118203.

[16] Y. Wang, H. Zhang, J. Zhang, C. Lu, Q. Huang, J. Wu, F. Liu, *J. Hazard. Mater.* **2011**, *192*, 35.

[17] S. Li, Z. Zhao, M. Liu, X. Liu, W. Huang, S. Sun, Y. Jiang, Y. Liu, J. Zhang, Z. Zhang, *Nano Energy* **2022**, *95*, 107031.

[18] C. Fu, T. Wu, G. Sun, G. Yin, C. Wang, G. Ran, Q. Song, *Appl. Catal. B Environ.* **2023**, *323*, 122196.

[19] C. Fu, M. Zhao, X. Chen, G. Sun, C. Wang, Q. Song, *Appl. Catal. B Environ.* **2023**, *332*, 122752.

[20] P. Wang, X. Li, S. Fan, X. Chen, M. Qin, D. Long, M. O. Tadé, S. Liu, *Appl. Catal. B Environ.* **2020**, *279*, 119340.

[21] X. Zhou, S. Wu, C. Li, F. Yan, H. Bai, B. Shen, H. Zeng, J. Zhai, *Nano Energy* **2019**, *66*, 104127.

[22] J. Wu, W. Wang, Y. Tian, C. Song, H. Qiu, H. Xue, *Nano Energy* **2020**, *77*, 105122.

[23] Q. Liu, D. Zhai, Z. Xiao, C. Tang, Q. Sun, C. R. Bowen, H. Luo, D. Zhang, *Nano Energy* **2022**, *92*, 106702.

[24] H. Zheng, X. Li, K. Zhu, P. Liang, M. Wu, Y. Rao, R. Jian, F. Shi, J. Wang, K. Yan, J. Liu, *Nano Energy* **2022**, *93*, 106831.

[25] M. Zhu, S. Li, H. Zhang, J. Gao, K. W. Kwok, Y. Jia, L.-B. Kong, W. Zhou, B. Peng, *Nano Energy* **2021**, *89*, 106474.

[26] X. Zhou, Q. Sun, D. Zhai, G. Xue, H. Luo, D. Zhang, *Nano Energy* **2021**, *84*, 105936.

[27] Q. Liu, Q. Hu, D. Zhai, Q. Sun, H. Luo, D. Zhang, *J. Mater. Chem. A* **2021**, *9*, 17841.

[28] X. Zhou, B. Shen, J. Zhai, N. Hedin, *Adv. Funct. Mater.* **2021**, *31*, 2009594.

[29] A. Zhang, Z. Liu, B. Xie, J. Lu, K. Guo, S. Ke, L. Shu, H. Fan, *Appl. Catal. B Environ.* **2020**, *279*, 119353.

[30] S. Li, Z. Zhao, D. Yu, J.-Z. Zhao, Y. Su, Y. Liu, Y. Lin, W. Liu, H. Xu, Z. Zhang, *Nano Energy* **2019**, *66*, 104083.

[31] X. Zhao, Y. Lei, P. Fang, H. Li, Q. Han, W. Hu, C. He, *Nano Energy* **2019**, *66*, 104168.

[32] P. Shen, P. Yin, Y. Zou, M. Li, N. Zhang, D. Tan, H. Zhao, Q. Li, R. Yang, B. Zou, B. Liu, *Adv. Mater.* **2023**, *35*, e2212172.
